# Kaixin Jieyu Granule attenuates neuroinflammation-induced depressive-like behavior through TLR4/PI3K/AKT/FOXO1 pathway: a study of network pharmacology and experimental validation

**DOI:** 10.1186/s12906-023-03970-5

**Published:** 2023-05-12

**Authors:** Manman Xu, Wujianwen Zhai, Ying Zhang, Juhua Pan, Jie Li, Shijing Huang

**Affiliations:** 1 Guang’ Anmen Hospital, Traditional Chinese Medicine Research and Development Center, China Academy of Chinese Medical Sciences, Beijing, 100053 China; 2 Department of oncology, Guang’ Anmen Hospital, China Academy of Chinese Medical Sciences, Beijing, 100053 China

**Keywords:** Kaixin Jieyu Granule, Depression, Network pharmacology, Traditional chinese medicine, Neuroinflammation

## Abstract

**Background:**

Kaixin Jieyu Granule (KJG), an improved formula of Kai-xin-san and Si-ni-san, is a highly effective formula with demonstrated efficacy in preventing depression in previous studies. However, the underlying molecular mechanisms of KJG’s antidepressant effects on inflammatory molecules remain unclear. This study aimed to explore the therapeutic effects of KJG on depression using network pharmacology and experimental validation.

**Methods:**

We employed a multi-faceted approach, combining high-performance liquid chromatography (HPLC), network pharmacology, and molecular docking, to unravel the underlying mechanisms of KJG’s anti-depressant effects. To confirm our findings, we conducted at least two independent in vivo experiments on mice, utilizing both the chronic unpredictable mild stress (CUMS)-induced and lipopolysaccharide (LPS)-induced models. Furthermore, the results of in vivo experiments were verified by in vitro assays. Behavioral tests were utilized to evaluate depression-like behaviors, while Nissl staining was used to assess morphological changes in the hippocampus. Pro-inflammatory cytokines and pathway-related protein expressions were determined using a combination of immunofluorescence staining, enzyme-linked immunosorbent assay (ELISA), and Western Blotting (WB).

**Results:**

Our network-based approaches indicated that ginsenoside Rg1 (GRg1) and saikosaponin d (Ssd) are the major constituents of KJG that exert an anti-depressant effect by regulating TLR4, PI3K, AKT1, and FOXO1 targets through the toll-like receptor, PI3K/AKT, and FoxO pathways. In vivo, KJG can attenuate depression-like behaviors, protect hippocampal neuronal cells, and reduce the production of pro-inflammatory mediators (TNF-α, IL-6, and IL-1β) by repressing TLR4 expression, which was regulated by the inhibition of FOXO1 through nuclear exportation. Furthermore, KJG increases the expression levels of PI3K, AKT, p-PI3K, p-AKT, and p-PTEN. Our in vitro assays are consistent with our in vivo studies. On the other hand, the above effects can be reversed by applying TAK242 and LY294002.

**Conclusion:**

Our findings suggest that KJG can exert anti-depressant effects by regulating neuroinflammation through the PI3K/AKT/FOXO1 pathway by suppressing TLR4 activation. The study’s findings reveal novel mechanisms underlying the anti-depressant effects of KJG, presenting promising avenues for the development of targeted therapeutic approaches for depression.

**Supplementary Information:**

The online version contains supplementary material available at 10.1186/s12906-023-03970-5.

## Introduction

Depression is a debilitating illness that poses a serious threat to human health, affecting an estimated 350 million individuals of all ages [1]. The underlying mechanisms linking depression are complex and potentially multifactorial, including dysregulation of cytokines, neurotransmitters, and hormonal systems [2]. Notably, inflammatory processes have been shown to be central to the pathogenesis of chronic conditions that may contribute to the onset of depression [3]. Toll-like receptor 4 (TLR4) is a crucial initiator of inflammatory responses that can be activated by lipopolysaccharide (LPS) and is involved in regulating stress responses [4, 5]. While standard antidepressant drugs are limited by side effects and a high rate of relapse [6], preclinical studies with traditional Chinese medicine (TCM) have shown impressive anti-depressant efficacy [7]. Network pharmacology has provided insight into the molecular mechanisms underlying TCM and has revealed multi-indication properties beyond its traditional applications [8].

In this context, we explore the therapeutic potential of Kaixin Jieyu Granule (KJG), an improved formula based on the Kai-xin-san [9, 10] and Si-ni-san [11, 12], for treating depression through network pharmacology and experimental validation. Animal experiments have demonstrated that Kai-xin-san can elicit an anti-depressant effect by stimulating neurogenesis and synaptic function through the PI3K/AKT pathway [9]. In addition to the PI3K/AKT inflammatory cascade, some protein candidates associated with susceptibility to stress-induced depression have been identified, including PTEN [10], FoxO1 [11], and NF-κB [12]. Clinical studies have indicated that Kai-xin-san [13, 14] and Si-ni-san [15, 16] have an apparent anti-depressant activity but limited effects when used alone. With years of clinical experience in treating cerebrovascular diseases, academician Yongyan Wang formulated the KJG for depression based on the classical prescriptions (Kai-xin-san and Si-ni-san). KJG is a combination of six traditional herbal extracts, including Renshen *(Panax ginseng C.A. Mey)*, Chaihu *(Radix Bupleuri)*, Chishao *(Radix Paeoniae Rubra)*, Fuling *(Poria Cocos(Schw.) Wolf.)*, Bajitian *(Morindae Officinalis Radix), and* Gancao *(licorice)* with the ratio of 3:5:5:5:3:3. After many years of clinical practice, our research group has confirmed that the effectiveness of KJG can reach 88.57% in patients with depression [17]. Besides, our previous studies [18–20] have suggested that KJG may alleviate depression-like behaviors by regulating cerebral cortical homeostasis, protecting hippocampal neurons, repairing white matter damage, and increasing cerebral blood flow supply in different animal stress models of depression.

Notably, neuroinflammation is a key factor in the onset of depression [21], with characteristic changes of neuroinflammation mainly manifested by microglial activation [22]. In chronic unpredictable mild stress (CUMS), microglia can secrete pro-inflammatory cytokines such as tumor necrosis factor-α (TNF-α), interleukin-6 (IL-6), and interleukin-1β (IL-1β), which induce neuroinflammation and aggravate depressive behavior [23]. Furthermore, numerous literature sources indicate that intraperitoneal injection of endotoxin LPS can trigger neuroinflammation with depressive-like behavior due to the activation of microglial [24]. To investigate the molecular events of neuroinflammation as related to its induction of depression-like behavior, the combination of CUMS-induced and LPS-induced mice models has been widely used [25]. However, the exact mechanism of depression accounting for inflammation remains obscure. Therefore, we need to explore the inflammatory mechanisms of KJG against depression and then extend its scope of clinical application. The study’s flowchart is depicted in Fig. S1.

## Materials and methods

### Drugs and reagents

Fluoxetine hydrochloride capsule (Flu) was purchased from Patheon France and was packaged by Lilly Suzhou Pharmaceutical Co., Ltd. Ginsenosides Rg1 (GRg1, B21057), and saikosaponin d (Ssd, B20150) were purchased from Yuanye Biotech (Shanghai, China). Lipopolysaccharide (LPS: L3129) was purchased from Sigma–Aldrich. A small-molecule-specific inhibitor of TLR4 signaling (TAK242, A3850) and PI3K/AKT inhibitor (LY294002, A8250) were from Apex Bio (Houston, Texas). TNF-α, IL-6, and IL-1β ELISA kits were purchased from BioProducts MD (Middletown, MD, USA). The antibodies to PI3K (ab178846), p-AKT (ab192623), AKT (ab179463), p-PTEN (ab76431), PTEN (ab170941), p-FOXO1 (ab131339), and FOXO1 (ab52857) were from Abcam, TLR4 antibody (AF7017) was from Affinity Biosciences, the p-PI3K-antibody (#4228) was from Cell signaling, the anti-β-actin (sc-47778) and goat anti-rabbit IgG (sc-516102) were from Santa Cruz Biotechnology.

### Animals

The animal experiment was conducted on male ICR mice (5 weeks, 24 g) from Sibeifu Beijing Biotechnology Co. Ltd. (Certificate No. 110324200103168645). These mice were individually housed in ventilated cages under controlled environmental conditions, including a temperature of 22 ± 3℃, a humidity of 45 ± 5%, and a 12-hour light-dark cycle. The experimental protocol adhered to the ARRIVE guidelines and was approved by the Ethics Committee of the China Academy of Chinese Medical Sciences at Guang’anmen Hospital (approval number MDL 2021-09-27-03).

### Preparation of KJG extract

The six herbs of KJG were purchased from the Guangan’men Hospital, China Academy of Chinese Medical Sciences, including Renshen *(Panax ginseng C.A. Mey)*, Chaihu *(Radix Bupleuri)*, Chishao *(Radix Paeoniae Rubra)*, Fuling *(Poria Cocos(Schw.) Wolf.)*, Bajitian *(Morindae Officinalis Radix), and* Gancao *(licorice)*. Renshen and Chaihu were extracted by adding 8 times 70% ethanol 3 times (each extraction period lasted 1.5 h), then filtered. The dregs were combined with the other four crude drugs (Chishao, Fuling, Bajitian, and Gancao), which were added 10 times the amount of water (1.3 times more for the first time) and extracted 2 times (each extraction period lasted 1.5 h), and then filtered. The alcoholic extract was concentrated at 60℃~70℃ under reduced pressure to a relative density of 1.08 ~ 1.12 (40℃~50℃), and the aqueous extract was concentrated at 70℃~80℃ under reduced pressure to a relative density of 1.22 ~ 1.26 (40℃~50℃). The alcoholic and aqueous extracts were combined and dried at 60℃ under vacuum to obtain dry extracts, which were crushed into a fine powder and set aside (Batch number: 20200914, 1000 mg extract drug/kg is equivalent to 3510 mg crude drug/kg). The constituents of KJG preparation were analyzed by HPLC, which was previously reported [19].

### Mechanism study of KJG intervention in depression by network pharmacology and molecular docking

#### Identify targets for KJG and depression

The TCMSP (http://ibts.hkbu.edu.hk/LSP/tc) and BATMAN database (https://bionet.ncpsb.org/batman-tcm/) were used to identify the chemical components of KJG. Afterward, the ingredients which meet the medicinal characteristics (OB ≥ 30% and DL ≥ 0.18) were screened out, a criterion suggested by the TCMSP database [26]. In the BATMAN database, “score ≥ 20” was utilized to screen the practical components [27]. Furthermore, the corresponding targets of KJG compounds were identified through a search of TCMSP and BATMAN databases.

To gain information about potential targets of depression, we took advantage of a series of public databases, including NCBI GEO [28] (accession code: GSE76826, GPL17077, 32 samples), GeneCards [29], OMIM [30], PharmGKB [31], TTD [32], and DrugBank (v3.0) [33], which high-throughput gene profiles and clinical data have been deposited by research institutes worldwide. After removing duplicate targets, the results were visualized using R ggplot2 (v.3.1.1) and Venny 2.1.

### Network construction

To identify shared genes between herbal compounds and diseases, Venny 2.1 software was used to perform an intersection analysis between the active compounds in KJG and the target genes associated with depression. A compound-target network was constructed, and Cytoscape 3.7.2 was further used to link the active ingredients with the intersected genes for data analysis. In addition, the overlapping genes were introduced into the STRING online database for protein-protein interaction (PPI) network analysis [34], and hub targets were selected using network topology analysis provided by CytoNCA. The results were ranked according to Eigenvector Centrality (EC), Degree Centrality (DC), Local average connectivity-based method (LAC), Closeness Centrality (CC), Betweenness Centrality (BC), and Network Centrality (NC).

### KEGG analysis and molecular docking

The Kyoto Encyclopedia of Genes and Genomes (KEGG) pathway analysis was performed using the DAVID and KEGG databases [35–37], and the results were further supported by molecular docking simulations. Specifically, the top 30 significantly enriched pathways were identified, and a KEGG scatter plot was generated [38]. Molecular docking simulations were carried out using Autodock Vina (version 1.1.2) to dock the crystal structure of the target with the chemical structures of the compounds downloaded from the PubChem database.

### Experimental methods

#### Establishment of the CUMS model

In this experiment, ten different stressors were applied to mice [39], including tail flicking (1 min), water deprivation (12 h), food deprivation (12 h), shaking (20 min), swimming in 4℃ cold water (5 min), damp sawdust (12 h), disruption of light cycle (24 h), physical restraint (2 h), cage tilt (24 h), and social crowding (12 h). These stimuli were applied randomly, while the control group of mice were left undisturbed in their home cages. After experiencing the CUMS for 5 weeks, all animals underwent sucrose preference training [40].

After that, the animals were divided into six groups (*n* = 10), namely: control (saline treatment), control + KJG8 (8000 mg crude drug/kg), CUMS (saline treatment), CUMS + Flu (3.33 mg/kg), CUMS + KJG4 (4000 mg crude drug/kg), and CUMS + KJG8 (8000 mg crude drug/kg). The dosages of KJG and Flu are calculated as follows: The KJG usage is 400 mg crude drug/kg/day (24000 mg/60kg body weight in adults daily) in patients with depression based on our previous clinical trials. The low dosage (KJG4; 4000 mg crude drug/kg) and high dosage (KJG8; 8000 mg crude drug/kg) in mice are similar at doses approximately 10 and 20 times higher than the adult clinical dosage [41, 42]. The Flu usage was 0.333 mg/kg/day (20 mg/60kg body weight in adults daily) in patients with depression, according to the manufacturer’s instruction. The dosage of Flu administered to mice (3.33 mg/kg) is approximately 10 times higher than the clinical dosage for adults. From week 6 to week 8, treatment groups were administered with saline, KJG, or Flu through daily gavage., during which the CUMS stimuli lasted throughout the procedure.

In another batch, the CUMS mice were randomly divided into four groups (*n* = 10), including control (saline treatment), CUMS (saline treatment), CUMS + KJG8 (8000 mg crude drug/kg), and CUMS + LY + KJG (8000 mg crude drug/kg). The dose and method of LY294002 (6 µg, intranasally) administration were determined in a previous study [43].

#### Establishment of the LPS model

The mice was randomly divided into four groups (*n* = 10), namely control (saline treatment), LPS (0.83 mg/kg, intraperitoneally), LPS + TAK242 (3 mg/kg, intraperitoneally), and LPS + KJG8 (8000 mg crude drug/kg). The mice were treated with TAK242 and KJG for 2 weeks once daily, during which LPS administration was commenced on day 10 for 4 consecutive days (day 10 to day 14). The dose and route of LPS and TAK242 administration were determined in a previous study [44, 45]. The flowchart of the experiments in vivo is illustrated in Fig. S2.

### Behavioral evaluation

The sucrose preference test (SPT) was conducted within 24 h of induction and drug administration, followed by the open field test (OFT), tail suspension test (TST), and forced swimming test (FST). The results of these tests were used as indicators of anorexia and as a measure of the correlation with depression [46]. For SPT, the mice were trained to adjust to drinking the sugar water 1% (W/V) two days before the experiment. After acclimation, SPT involved the use of two bottles, one containing 1% sucrose solution and the other pure water, with the amount of sucrose intake measured and recorded by weighing (a 12-h period). OFT measured the movement of mice in a uniformly lit, open black square box (50 cm × 50 cm × 40 cm) for 6 min. Following a 2-minute observation period, a digital recording was taken for the subsequent 4 minutes to capture the total number of platform crossings between quadrants and the time spent in each quadrant. TST measured the immobility time of mice in an inverted position, while FST measured the immobility time of mice in water. For TST, the quantification of total immobility time was carried out over a 6-minute duration after 2 min of acclimatization. Specifically, total immobility time was defined as the period during which the body remained in a vertical, upside-down position without any discernible struggling, in accordance with the criteria outlined in reference [47]. During the FST, the duration of immobility time, which was defined as the period in which the mouse maintained its head above the water by making slight but necessary movements, was measured over the final 6 minutes of the test, in accordance with the criteria outlined in reference [48].

### Immunofluorescence and nissl staining

For brain tissue immunofluorescence, the samples were sequentially immersed in 95%, 85%, and 75% ethanol for deparaffinization and washed for 5 min with distilled water. Following the addition of 10% goat serum, TLR4 antibody (diluted 1/500) was incubated on the sections in blocking buffer overnight at 4°C. Subsequently, the samples were subjected to secondary antibody incubation for 1 h at 37°C and nuclear staining, and finally analyzed using a fluorescence microscope (DM3000, Leica). For Nissl staining, the 5 µm samples were deparaffinized and rehydrated through xylene and gradient alcohol according to the above methods. Next, the sections were embedded with toluidine blu (1%) for 20 min at 50°C. After completion of staining, the slices were rinsed twice with distilled water and then dehydrated using 95% ethanol to remove excess moisture from the samples. Finally, resultant sections were obtained by immersing in xylene and sealing with neutral gum, which can be observed under a light microscope and quantified by IPP 6.

### Cell culture and treatments

BV2 microglia (murine microglial cell line) were cultivated in DMEM medium (Gibco) containing 15% fetal bovine serum (Gibco), 2 mM l-glutamine (Sigma), and 1% penicillin/streptomycin (Sigma). BV2 microglial cells were plated at a 2 × 10^5^ /ml seeding density for all experiments. Before 24 h incubation with LPS (1 µg/mL), the cells were administrated with the main compounds of KJG (GRg1, 225 µM; Ssd, 35 µM) and LY294002 (10 µM) for 1 h, respectively. The doses of GRg1, Ssd, LY294002, and LPS were selected according to the results of Cell Counting Kit-8 (CCK8, Sigma; Table S1 and 2) and previous studies [49, 50]. The flowchart of the experiments in vitro is illustrated in Fig. S3.

### Immunocytochemistry

BV2 cells were initially washed with PBS and fixed with 4% PFA. Subsequently, blocking was performed using 5% goat serum, followed by overnight incubation with TLR4 (diluted 1/500) and FOXO1(diluted 1/1000) antibodies at 4℃. After washing the cells twice with PBS, secondary antibodies were applied and incubated at 37℃ for one hour. Finally, DAPI was used for nuclear staining of the BV2 cells, which were analyzed under a fluorescence microscope (DM3000, Leica).

### Enzyme-linked immunosorbent assay (ELISA)

Mice were euthanized by increasing carbon dioxide concentrations, followed by cervical dislocation. The collected blood samples were centrifuged (at 3500 rpm for 15 minutes) to obtain serum for subsequent analysis. The levels of TNF-α, IL-6, and IL-1β in the serum were measured using enzyme-linked immunosorbent assay (ELISA). The samples were detected and quantified using an ELISA reader, with the absorbance set at 450 nm, to obtain quantitative results for these indicators.

### Western blot (WB) detection

Prior to protein extraction, collected samples were homogenized and lysed to obtain supernatant for subsequent protein extraction. A 10% SDS-PAGE gel was prepared, and protein samples were added to each well for electrophoresis separation. The separated protein bands were transferred onto a PVDF membrane and washed with a series of solutions, including 100% methanol, transfer buffer, and distilled water, to remove impurities and ions that may remain on the membrane. Primary antibodies were added to the membrane and incubated overnight at 4℃ to ensure sufficient time for protein-antibody binding, followed by incubation with secondary antibody in the dark for 1 hour with gentle shaking. Finally, ECL substrate was added, and the membrane was placed into a chemiluminescence imaging system to detect the expression level of the protein. The primary antibodies of anti-TLR4 and anti-p-AKT for WB were used at a dilution of 1/500, and the other primary antibodies were used at a dilution of 1/1000.

### Statistical analysis

one-way analysis of variance (ANOVA) was used to measure differences among multiple groups (≥ 2), followed by Sidak or Turkey’s multiple comparison test for further analysis. All results were expressed as means and standard errors of the mean (SEM), unless otherwise indicated, with a significance level of *P* < 0.05. Statistical analysis was conducted using GraphPad Prism software (version 6.01).

## Results

### The potential targets of KJG and depression

During the preliminary screening, 191 compounds present in KJG were initially identified as candidates, with 55 originating from Renshen, 41 from Chaihu, 4 from Bajitian, 16 from Fuling, 14 from Chicago, and 74 from Gancao. Building upon previous HPLC findings [19], 9 additional compounds were added to this dataset, including nistose, paeoniflorin, saikosaponin A, saikosaponin D, glycyrrhizic acid Ammonium salt, liquiritin, ginsenoside Rg1, ginsenoside Re and ginsenoside Rb1 (Table S3). Moreover, a total of 6074 targets were obtained from database searches, with 2118 associated with Renshen, 2967 with Chaihu, 108 with Bajitian, 23 with Fuling, 136 with Chishao, and 722 with Gancao. To ensure accurate results, 2732 targets of KJG were selected by setting the “score value” to “20” and removing duplicates.

**Fig. 1:**
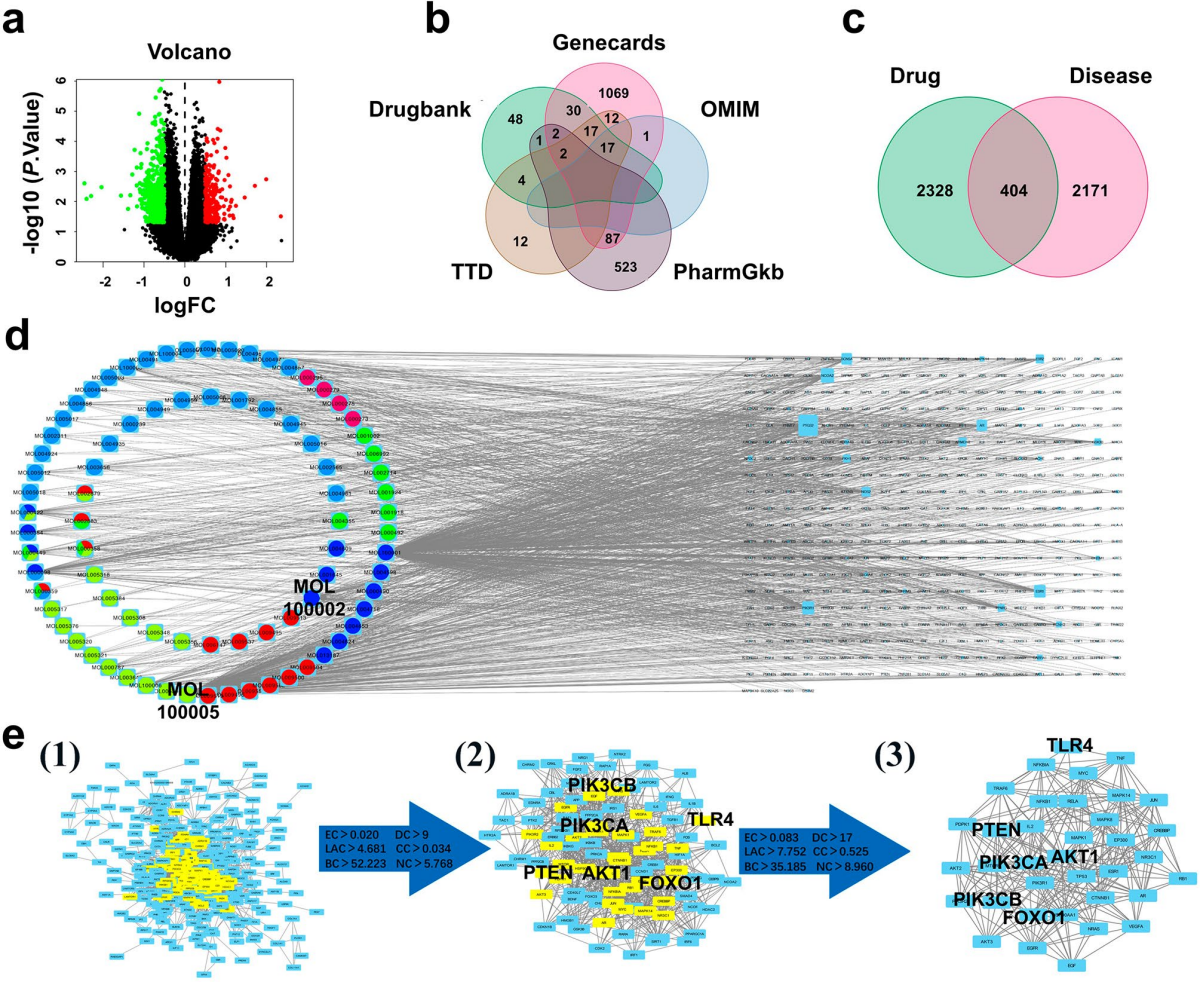
Identification of potential compounds and targets in KJG against depression. (**a**) The volcano distributions of differentially expressed genes (cut-off of *p*value = 0.05). The abscissa represents the log2 fold change value, and the ordinate represents the adjusted *P*-value (-log10 *P*-value). The green and red dots represent up-regulated and down-regulated genes in depression, respectively. (**b**) Venn map: The target genes of depression were obtained by combining the five databases. (**c**) Venn Diagram. The interaction of KJG targets and depression targets. (**d**) Compound-target network: The blue rectangles represent overlapping genes between KJG and depression; the light green, dark blue, dark green, magenta, red, and navy blue ovals represent the compounds from Renshen, Chaihu, Chishao, Fuling, and Gancao, respectively. (**e**) PPI network: (1) The interactions among the common targets from the STRING database; (2) The first filtered network was extracted from (1) by the EC, DC, LAC, CC, BC, and NC medium; (3) The second filtered network was extracted from (2) by the EC, DC, LAC, CC, BC, and NC medium

Subsequently, the GEO database was queried for depression-related differential gene expression. In the volcano plot generated by R ggplot2 (v.3.1.1), 808 DEGs were identified, with 536 genes exhibiting down-regulation and 272 genes exhibiting up-regulation (Fig. 1a). Additionally, 1987 candidate targets involved in depression regulation were obtained through five databases, including 1220 from GeneCards, 1 from OMIM, 615 from PharmGkb, 47 from Therapeutic Targets, and 104 from DrugBank. The Venn diagram of the predicted targets from these databases is presented in (Fig. 1b), and after removing duplicates, 2575 depression-related targets were obtained.

### Network construction analysis

The identification of overlapping genes between KJG targets and depression-associated genes was performed using Venny Software, resulting in 404 common targets (Fig. 1c). A compound-target network was subsequently constructed and visualized using Cytoscape 3.7.1 software based on the common targets, which contained 486 nodes and 645 edges, with 82 active compounds and 404 targets (Fig. 1d). Their topological score in the network determined the size of the targets, and nodes were ranked according to their degree value. MOL100005 ginsenoside Rg1 (GRg1, PubChem: 441923) in Renshen and MOL100002 saikosaponin d (Ssd, PubChem: 107793) in Chaihu were found to be the essential compounds in KJG against depression.

To elucidate the interactions among the common targets, a PPI network was constructed using the STRING database (Fig. 1e). The median parameters of EC, DC, LAC, CC, BC, and NC were calculated for the first and second pictures, which were 0.02, 9, 4.681, 0,034, 52.223, and 5.768 and 0.083, 17, 7.752, 0,525, 35.185, and 8.960, respectively. A total of 36 key targets were extracted from the third picture according to twice the median of the six parameters, which were likely involved in the anti-depressant mechanisms of KJG through direct and indirect effects [51].

### KEGG and molecular docking analysis

**Fig. 2:**
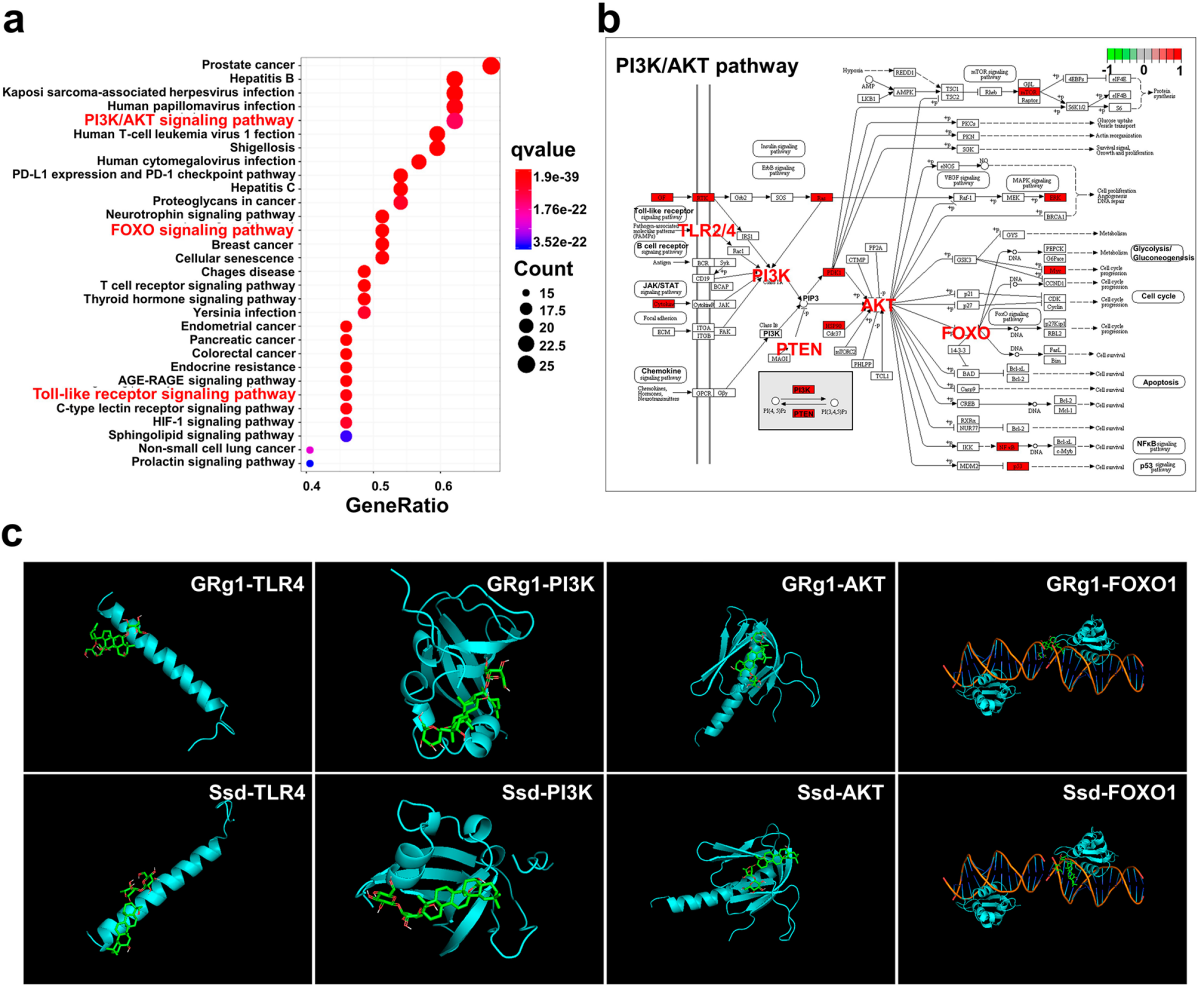
KEGG analysis and molecular docking simulation. (**a**) The top 30 KEGG-enriched terms were screened (cut-off of *q*value = 0.05). The abscissa represents the GeneRatio, the ratio of genes enriched in one KEGG pathway to the number of genes in all KEGG pathways. The ordinate represents the pathway name. The spot size means the degree of enrichment, and the color represents the *q*-value of each path. (**b**) Map of PI3K/AKT pathway. The critical genes of KJG in treating depression were labeled red on the map. (**c**) Molecular docking simulation for GRg1 and Ssd interactions with TLR4, PI3K, AKT, and FOXO1.

In this study, 36 critical targets were filtered from the PPI network and subsequently subjected to KEGG analysis. The top 30 enrichment terms, including PI3K/AKT, FoxO, Toll-like receptor, and cancer pathways, were identified and presented in Fig. 2a. Furthermore, we rendered a KEGG map using the path view package in R, highlighting the enriched genes in the PI3K/AKT signaling pathway (Fig. 2b). Analysis of Table S4 revealed that TLR4 and FOXO1 were common genes in the enriched PI3K/AKT and FoxO signaling pathways, and were critical genes in the KEGG map. Based on these findings, we propose that KJG may exert anti-depressant effects by relieving inflammation through the PI3K/AKT/FOXO1 pathway, which is potentially regulated by TLR4.

To validate the network pharmacology results, molecular docking was conducted between the identified essential genes (TLR4, PI3K, AKT, and FOXO1) and active compounds (GRg1 and Ssd) in KJG. The docking structure with the highest score was selected based on its excellent quality and high stability [52]. The affinity score presented in Table 1 indicates that GRg1 and Ssd could fit well into the binding site of TLR4, PI3K, AKT, and FOXO1 in the molecular docking simulation. Among the 8 output docking poses (Fig. 2c), Ssd with FOXO1 and GRg1 with FOXO1 were found to have the highest docking score. Overall, the findings suggest that KJG may have therapeutic potential for depression by targeting the PI3K/AKT/FOXO1 pathway and its potential regulation by TLR4. However, further experimental validation is needed to confirm these findings.

**Table 1:** Results of docking score values of compounds to their candidate molecular targets

Compounds	Docking Score (kcal/mol)			
	TLR4	PI3K	AKT	FOXO1
GRg1	-6.7	-6.5	-6.0	-8.9
Ssd	-6.8	-7.5	-8.4	-9.9

### KJG ameliorated depressive behavior by reducing neuroinflammation response in CUMS-induced mice

**Fig. 3:**
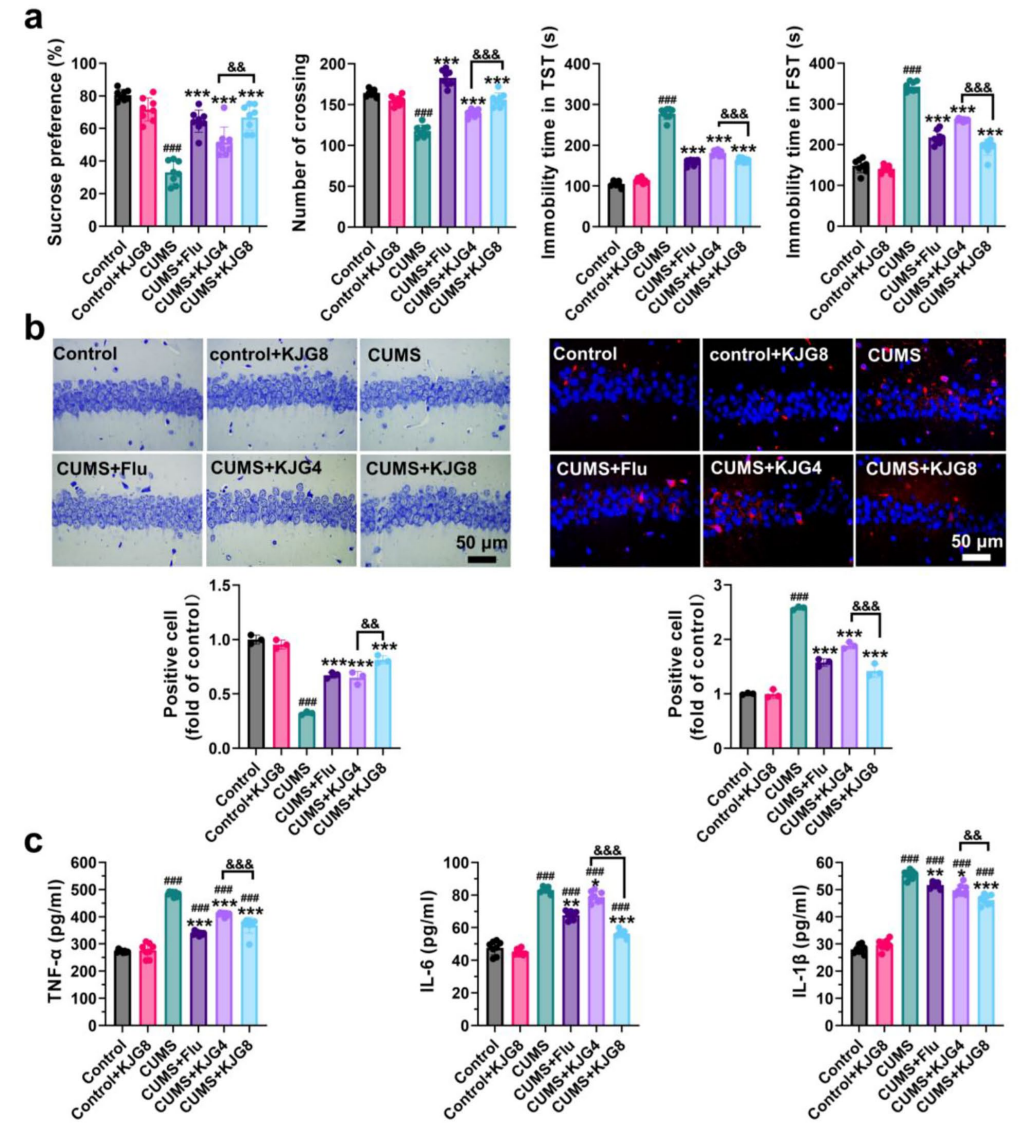
KJG ameliorates depressive behavior by reducing neuroinflammation in CUMS-induced mice. (**a**) The effect of KJG on the depressive behaviors induced by CUMS in mice (5 weeks, *n* = 10). The sucrose preference test (SPT, first), the open field test (OFT, second), the Tail suspension test (TST, third), and the forced swimming test (FST, fourth). (**b**) The morphological changes of CA1 region in hippocampal tissue (*n* = 3). Nissl staining (the left), and immunofluorescence staining of TLR4 and their statistical analysis (the right) (Red, TLR4; blue, DAPI; Scale bar: 50 μm). (**c**) The levels of TNF-α, IL-6, and IL-1β in the serum of mice and their statistical analysis (*n* = 8). One-way ANOVA: # *P* < 0.05, ## *P* < 0.01, ### *P* < 0.001 vs. control group; * *P* < 0.05, ** *P* < 0.01, *** *P* < 0.001 vs. CUMS group; & *P* < 0.05, && *P* < 0.01, &&& *P* < 0.001, vs. CUMS + KJG8 group. KJG4, 4000 mg crude drug/kg; KJG8, 8000 mg crude drug/kg

The present study aimed to investigate the therapeutic mechanism of KJG in depression. To achieve this, behavioral tests were performed on CUMS-induced depressive mice (Fig. 3a). In the SPT, the CUMS group displayed reduced sucrose consumption compared to the control group (*P* < 0.001, *F* = 2.374). However, treatment with KJG and Flu significantly inhibited the reduction in sucrose consumption. In the OFT, the CUMS group exhibited lower locomotor activity than the control group (*P* < 0.001, *F* = 2.374), whereas treatment with KJG (*P* < 0.001) and Flu (*P* < 0.001) significantly improved the locomotor activities of the mice. In the FST and TST, the immobility time of the CUMS group was significantly longer than that of the control group (*P* < 0.001, F = 1.420 and *P* < 0.001, F = 1.507, respectively). However, treatment with KJG (4000 mg crude drug/kg, *P* < 0.001; 8000 mg crude drug/kg, *P* < 0.001) and Flu (*P* < 0.001) significantly reversed the extension of immobility time when compared to the model group. Notably, a significant difference (*P* < 0.001) between the KJG4 and KJG8 groups suggested that higher doses of KJG have better antidepressant effects.

To explore the pathological mechanisms of KJG in treating depression, we performed Nissl staining on the hippocampal cells in the CA1 area of mice (Fig. 3b, left). The results showed that the hippocampal cells of the CUMS group were damaged, with fewer cells, loose arrangement, pyknosis of the nucleus, and disintegration of the Nissl body (*P* < 0.001, F = 0.3795). However, treatment with KJG (8000 mg crude drug/kg, *P* < 0.001) and Flu (*P* < 0.001) dramatically alleviated neuronal injury and significantly increased the number of Nissl bodies. To ensure the role of TLR4 in the pathological process of depression, localization of TLR4 was observed in Fig. 3b (immunofluorescent staining, the right). The expression of TLR4 was increased in the CUMS group compared with the control group (*P* < 0.001, F = 0.4112). However, treatment with KJG (8000 mg crude drug/kg, *P* < 0.001) and Flu (*P* < 0.001) remarkably reduced the expression of TLR4.

Furthermore, we investigated the relationship between pro-inflammatory cytokines and depression by measuring the concentration of TNF-α, IL-6, and IL-1β in mice (Fig. 3c). The levels of TNF-α (*P* < 0.001, F = 2.374), IL-1β (*P* < 0.001, F = 0.4534), and IL-6 (*P* < 0.001, F = 1.753) were significantly increased in the CUMS group compared to the control group. However, significantly decreased levels of proinflammation factors were present in groups with KJG (*P* < 0.001) and Flu (*P* < 0.001) compared with those untreated mice. The data proved that the neuroinflammation in mice induced by CUMS could be repressed by KJG via downregulating the proinflammation factors.

Of note, the potential toxicity of KJG particularly needs to be considered and evaluated. As shown in Fig. 3, no significant differences were observed in the behavioral tests, Nissl staining, TLR4 staining, and pro-inflammatory cytokines levels between the control and control + KJG8, suggesting that KJG has no neurotoxicity and excitability to normal animals.

### KJG reduced neuroinflammation response by inhibiting TLR4 in LPS-induced mice

**Fig. 4:**
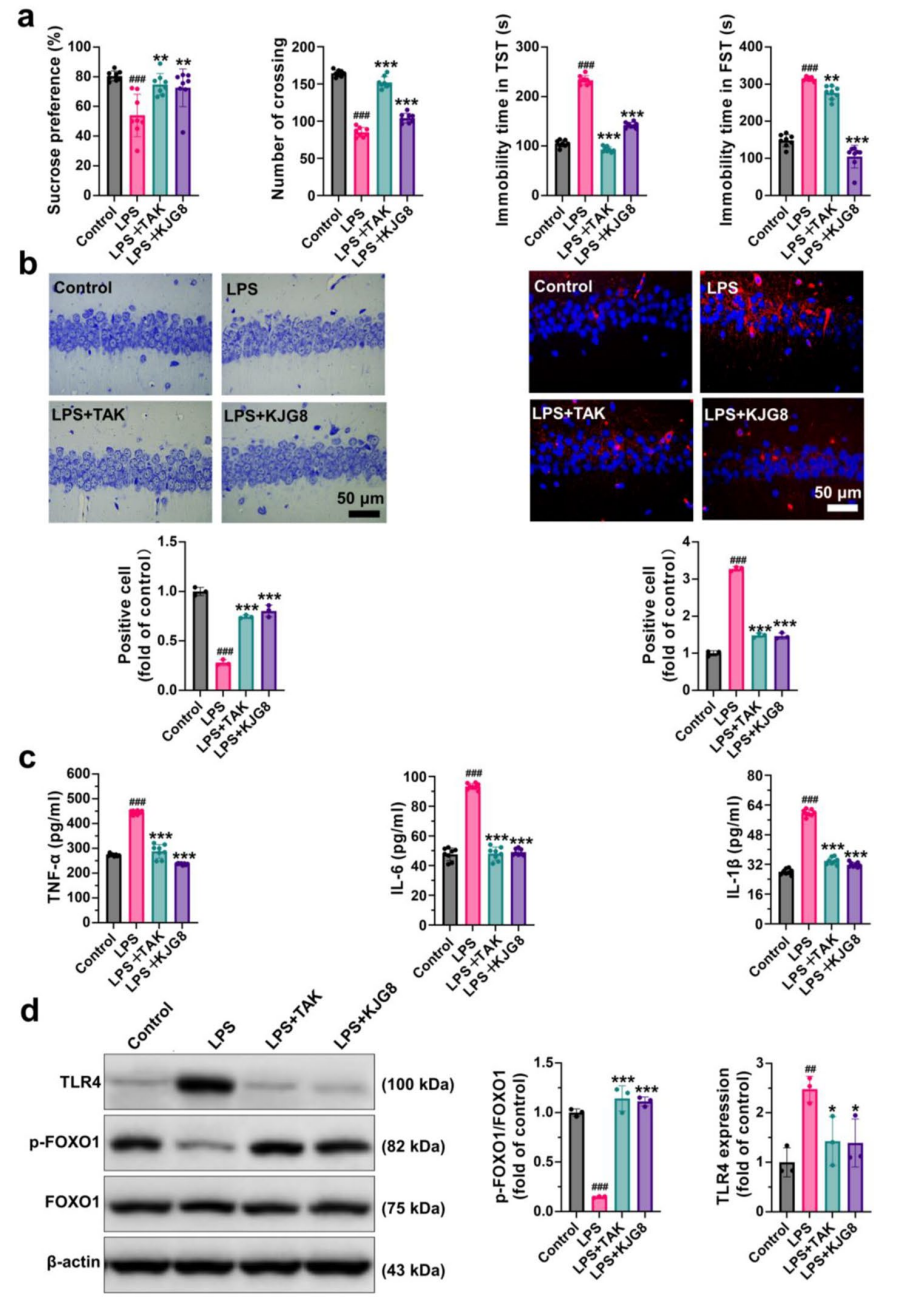
KJG reduces neuroinflammation response by inhibiting TLR4 in LPS-induced mice. (**a**) The effect of KJG on the depressive behaviors induced by LPS in mice (5 weeks, *n* = 10). The sucrose preference test (SPT, first), the open field test (OFT, second), the Tail suspension test (TST, third), and the forced swimming test (FST, fourth). (**b**) Nissl staining and immunohistochemistry staining were performed after behavior tests (*n* = 3; Scale bar: 50 μm). (**c**) The levels of TNF-α, IL-6, and IL-1β in the serum of mice and their statistical analysis (*n* = 8). (**d**) WB analysis for TLR4, p-FOXO1, and FOXO1 in hippocampal tissues (*n* = 3). One-way ANOVA: # *P* < 0.05, ## *P* < 0.01, ### *P* < 0.001 vs. control group; * *P* < 0.05, ** *P* < 0.01, *** *P* < 0.001 vs. LPS group

LPS and TAK242 were employed to investigate the mechanism underlying the antidepressant action of KJG mediated by TLR4. As shown in Fig. 4a, LPS administration induced depression-like behavior, which may be associated with TLR4 activation. Specifically, sugar water consumption (*P* < 0.001, F = 1.348) and exercise activity (*P* < 0.001, F = 0.6100) were significantly decreased in LPS-injected mice compared to the control group, while the immobility time in the TST (*P* < 0.001, F = 0.6515) and FST (*P* < 0.001, F = 0.8591) was significantly increased. By contrast, treatment with KJG and TAK242 increased sucrose preference, crossing numbers, and reduced immobility time compared to the LPS group (*P* < 0.01).

The Nissl staining analysis results (Fig. 4b, left) showed that LPS-induced neuroinflammation led to loosely arranged hippocampal CA1 neural cells and a lower number of Nissl body counts (*P* < 0.001, F = 0.7218) compared to the control group. In contrast, KJG and TAK242 treatments resulted in marked preservation of the number of Nissl-positive stained neurons compared with the LPS group (*P* < 0.001), indicating a neuroprotective effect of KJG and TAK242 against inflammation in hippocampal neuronal cells. Immunohistochemistry staining (Fig. 4b, right) demonstrated that LPS caused TLR4 overexpression (*P* < 0.001, F = 0.7457), whereas TAK242 and KJG significantly inhibited the action of TLR4 (*P* < 0.001), indicating that KJG and TAK242 could reduce neuroinflammation by blocking the TLR4 pathway.

The increase in pro-inflammatory cytokines is a hallmark of TLR4 activation [53, 54]. As presented in Fig. 4c, with the concomitant rise of TLR4, LPS administration led to a significant increase in the levels of TNF-α (*P* < 0.001, F = 9.126), IL-1β (*P* < 0.001, F = 0.3495), and IL-6 (*P* < 0.001, F = 2.043). However, TAK242 injection markedly prevented the elevation of pro-inflammatory cytokines (*P* < 0.001) due to its strong inhibitory effect on TLR4 expression. The KJG group presented the same level of pro-inflammatory cytokines as the TAK242 group.

The literature has shown that FOXO1-binding sites are localized on the TLR4 gene, indicating that the transcription of TLR4 is regulated by FOXO1 [55]. WB results (Fig. 4d) demonstrated that TLR4 and FOXO1 expression in the hippocampus was enhanced in LPS-injected mice compared to the control group (TLR4, *P* < 0.01, F = 0.1919; FOXO1, *P* < 0.01, F = 0.9073), whereas the phosphorylation of FOXO1 (*P* < 0.001) was downregulated. Notably, the TAK242 and KJG groups exhibited several similarities, such as the consistent trend of TLR4 downregulation and p-FOXO1 upregulation (*P* < 0.05). Collectively, these results suggest that a high level of TLR4 is accompanied by a decrease in p-FOXO1 and an increase in FOXO1 expression. Thus, TLR4 drives neuroinflammation pathogenesis and exacerbates depressive behaviors, but the administration of KJG and TAK242 can reverse this phenomenon through the vital role played by FOXO1.

### KJG reduced neuroinflammation response by inhibiting TLR4 via PI3K/AKT pathway in CUMS-induced mice

**Fig. 5:**
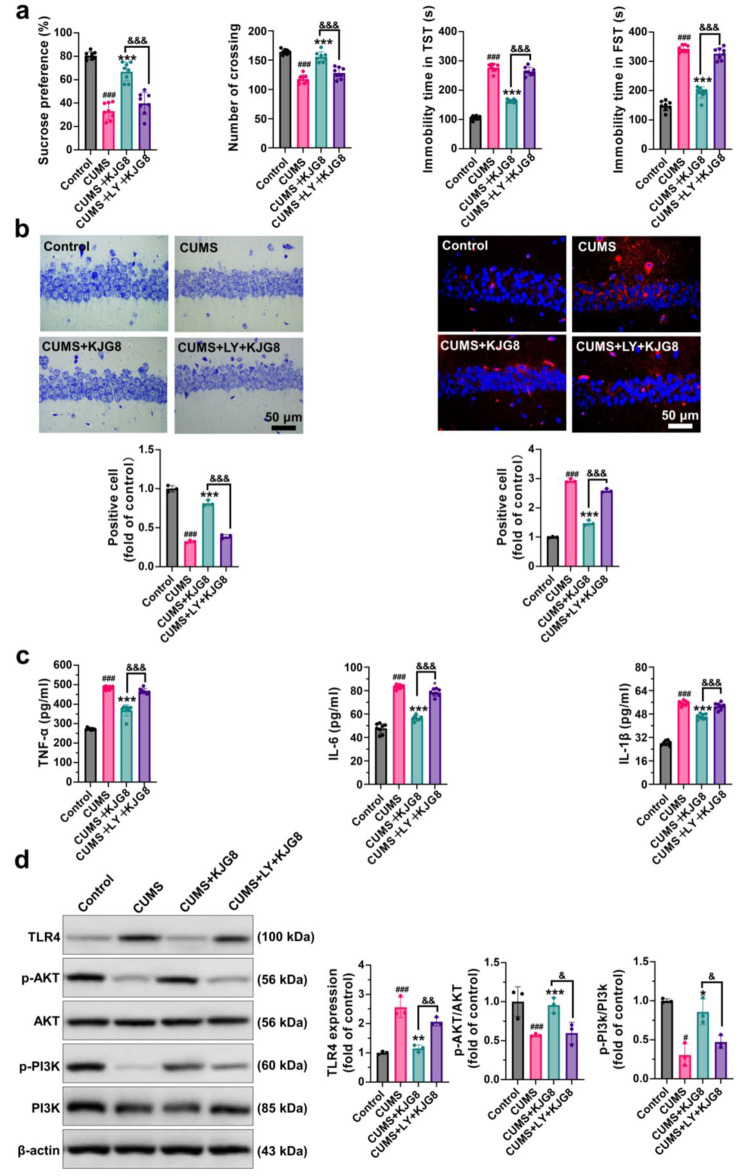
KJG reduces neuroinflammation response by inhibiting TLR4 via the PI3K/AKT pathway in CUMS-induced mice. (**a**) The SPT, OFT, TST, and FST tests were conducted to examine the depression-like state (*n* = 10). (**b**) Nissl staining and immunohistochemistry staining were performed after behavior tests (*n* = 3; Scale bar: 50 μm). (**c**) The levels of TNF-α, IL-6, and IL-1β in the serum of mice and their statistical analysis (*n* = 8). (**d**) TLR4, p-AKT, AKT, p-PI3K, and PI3K in hippocampal tissues were detected by WB. (*n* = 3). One-way ANOVA: # *P* < 0.05, ## *P* < 0.01, ### *P* < 0.001 vs. control group; * *P* < 0.05, ** *P* < 0.01, *** *P* < 0.001 vs. CUMS group; & *P* < 0.05, && *P* < 0.01, &&& *P* < 0.001 vs. CUMS + LY + KJG8 group

The involvement of the PI3K/AKT pathway in the expression of proinflammatory cytokines and TLR4 has been reported in the literature [56, 57]. To investigate whether TLR4 is regulated by the PI3K/AKT pathway, LY294002 was employed to explore their association. In SPT and OFT (as shown in Fig. 5a), the consumption of sucrose was significantly lower in the LY294002 + KJG group (*P* < 0.001, F = 1.777), and the locomotor activity was weaker than in the KJG group (*P* < 0.001, F = 2.303). Moreover, the immobility time in TST and FST was longer (TST, *P* < 0.001, F = 2.006; FST, *P* < 0.05, F = 0.4905). However, there were no significant differences between KJG + LY294002 and CUMS group, suggesting that LY294002 dramatically reversed the anti-depressive efficacy of KJG. Furthermore, as demonstrated in Fig. 5c, the recruitment of TNF-α, IL-6, and IL-1β was significantly increased in the KJG + LY294002 group compared to the KJG group, indicating that LY294002 blocked the anti-inflammatory effects of KJG. As revealed by Nissl staining in Fig. 5b (the left), neuronal cells in the LY294002 + KJG group were arranged loosely or missing, and Nissl bodies were lightly stained or even dissolved compared to those in the KJG group (*P* < 0.001, F = 0.3363). Immunohistochemistry staining (Fig. 5b, the right) showed that animals treated with KJG (*P* < 0.01, F = 1.022) exhibited better TLR4 inhibition than those in the CUMS and LY294002 + KJG groups. Additionally, TLR4 expression in the neural cells of the hippocampus increased under the exposure of LY294002, followed by decreased neural cells.

Furthermore, WB results depicted in Fig. 5d showed that after TLR4 overexpression (*P* < 0.001, F = 0.4505), the phosphorylation of PI3K (*P* < 0.05, F = 0.7480) and AKT (*P* < 0.001, F = 0.4774) was significantly reduced in the CUMS group compared to the control. However, KJG enhanced the ratio of p/t-PI3K and p/t-AKT expressions (*P* < 0.05), which in turn limited the TLR4 expression (*P* < 0.01) after stimulation with CUMS. Additionally, blocking PI3K activity by LY294002 significantly attenuated the amplified activities of p-AKT and p-PI3K elicited by KJG (*P* < 0.05). These results suggested that the intense neuroinflammation response from TLR4 overexpression was negatively modulated by the PI3K/AKT pathway triggered by KJG.

### KJG ameliorated TLR4-induced neuroinflammation in CUMS mice via PI3K/AKT-FOXO1 pathway

**Fig. 6:**
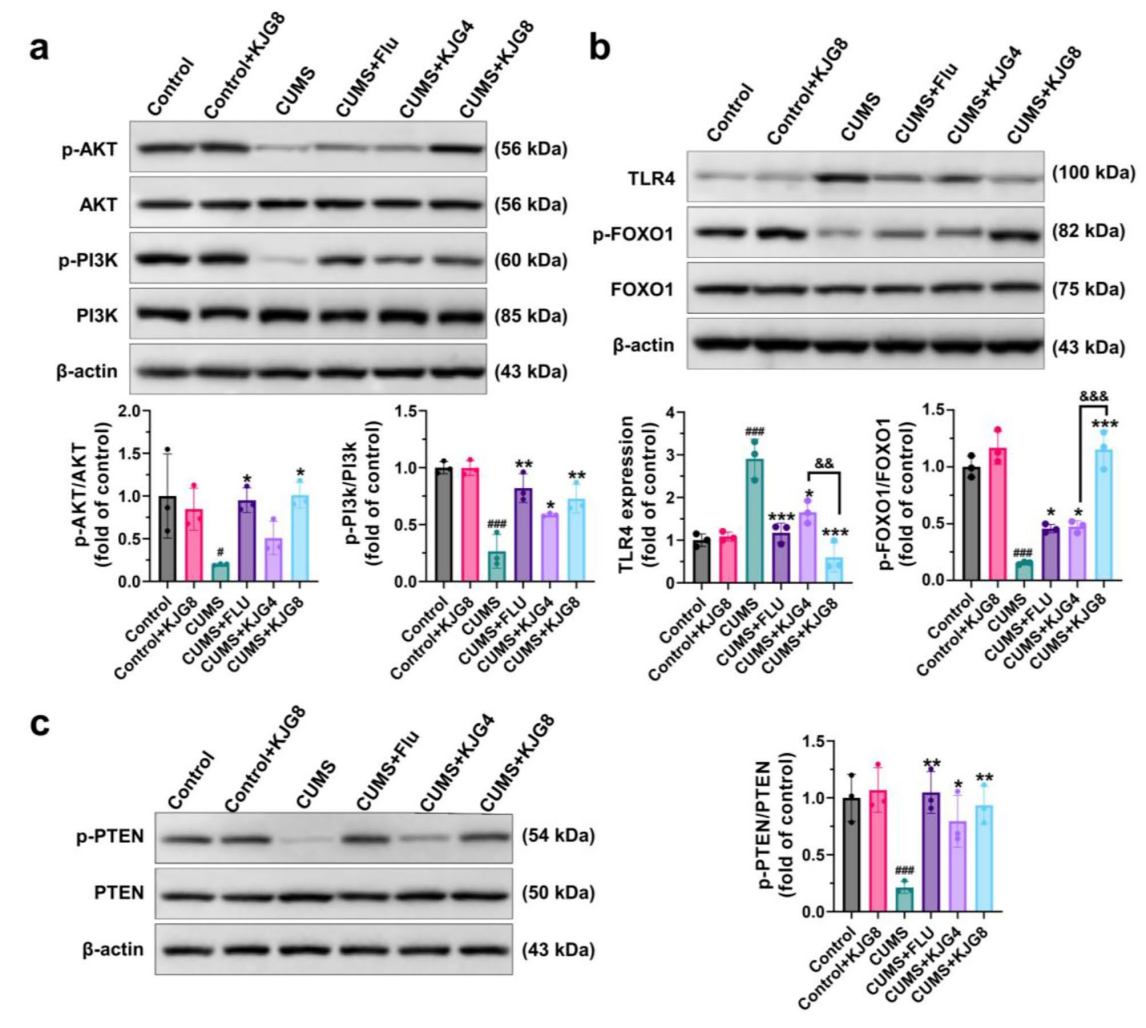
KJG ameliorates TLR4-induced neuroinflammation in CUMS mice via the PI3K/AKT/FOXO1 pathway. WB analysis for TLR4, p-PI3K, PI3K, p-AKT, AKT, p-FOXO1, FOXO1, PTEN, and p-PTEN in hippocampal tissues (*n* = 3). One-way ANOVA or unpaired t-tests: # *P* < 0.05, ## *P* < 0.01, ### *P* < 0.001 vs. control group; * *P* < 0.05, ** *P* < 0.01, *** *P* < 0.001 vs. CUMS group; & *P* < 0.05, && *P* < 0.01, &&& *P* < 0.001, vs. CUMS + KJG8 group

To investigate the underlying mechanism of KJG on depression, the proteins involved in the PI3K/AKT/FOXO1 pathway were examined by WB. Compared with the control group, the expressions of p-AKT (*P* < 0.05, F = 0.8603), p-PI3K (*P* < 0.001, F = 0.4608), p-FOXO1 (*P* < 0.001, F = 0.7388), and p-PTEN (*P* < 0.001, F = 0.2358) were downregulated in hippocampal tissues of mice exposed to CUMS, while TLR4 (*P* < 0.001, F = 1.123) was upregulated (Fig. 6a-c). Furthermore, treatment with Flu (*P* < 0.05) and KJG (8000 mg crude drug/kg, *P* < 0.05) significantly upregulated the levels of p-AKT, p-PI3K, p-FOXO1, and p-PTEN, and restrained TLR4 (*P* < 0.001) compared with CUMS. Notably, a slight increase in the expression of p-FOXO1 was observed in the Flu and KJG (4000 mg crude drug/kg) groups compared with the CUMS group (Fig. 6b). These results indicate that KJG may exert its anti-depressive effects by regulating the PI3K/AKT/FOXO1 pathway, which is involved in regulating TLR4 expression.

### The main compounds of KJG reduced neuroinflammation response by inhibiting TLR4 via PI3K/AKT-FOXO1 pathway in LPS-induced BV2 cells

**Fig. 7:**
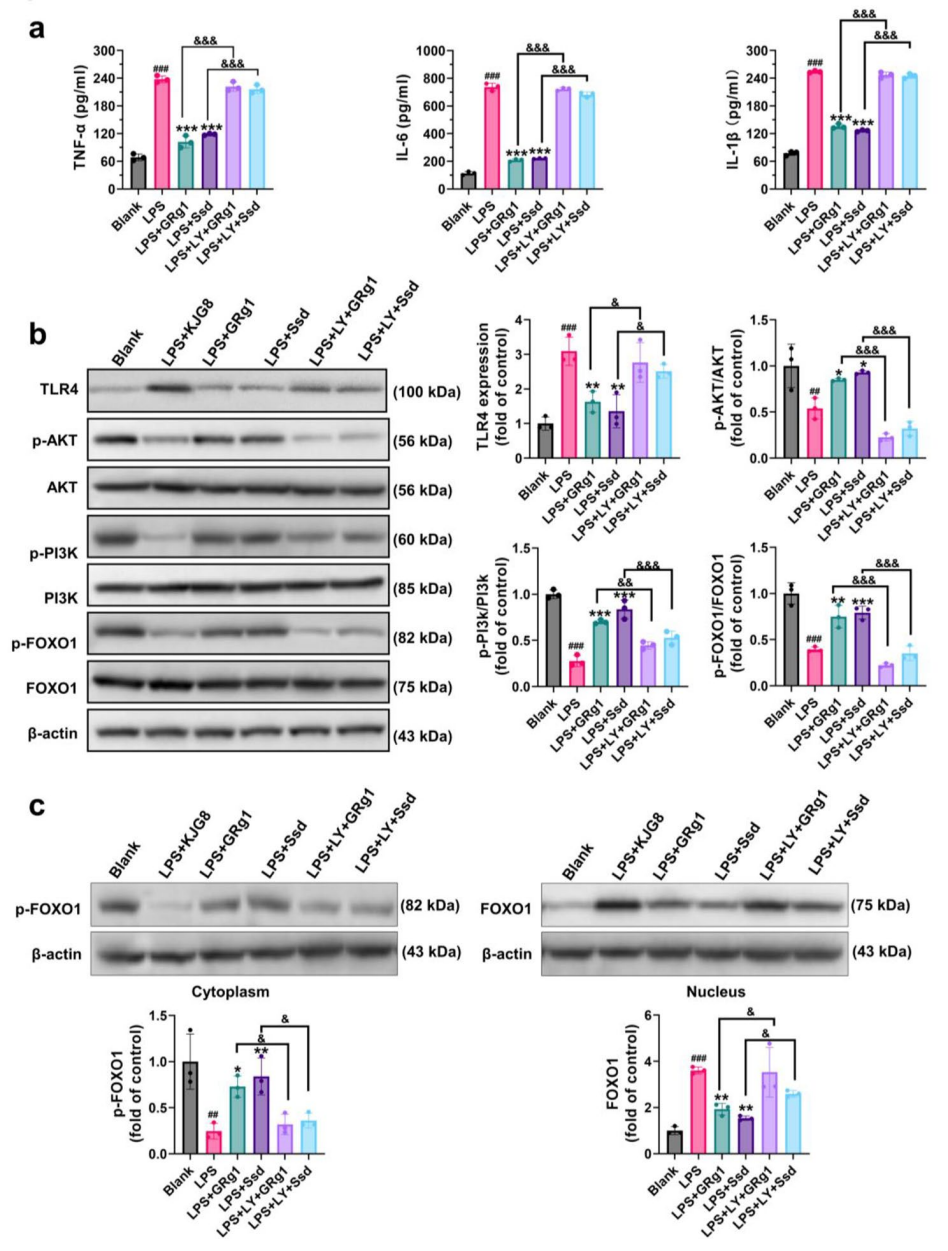
The main compounds of KJG (GRg1 and Ssd) reduced neuroinflammation response by inhibiting TLR4 via PI3K/AKT/FOXO1 pathway in LPS-induced BV2 cells. (**a**) The levels of TNF-α, IL-6, and IL-1β in BV2 cells and their statistical analysis (*n* = 3). (**b**) WB analysis for TLR4, p-PI3K, PI3K, p-AKT, AKT, p-FOXO1, and FOXO1in BV2 cells (*n* = 3). (**c**) WB analysis for FOXO1 in nucleus and cytoplasm of BV2 cells (*n* = 3). One-way ANOVA: # *P* < 0.05, ## *P* < 0.01, ### *P* < 0.001 vs. blank group; * *P* < 0.05, ** *P* < 0.01, *** *P* < 0.001 vs. LPS group. & *P* < 0.05, && *P* < 0.01, &&& *P* < 0.001 vs. LPS + LY + GRg1 group or LPS + LY + Ssd group

**Fig. 8:**
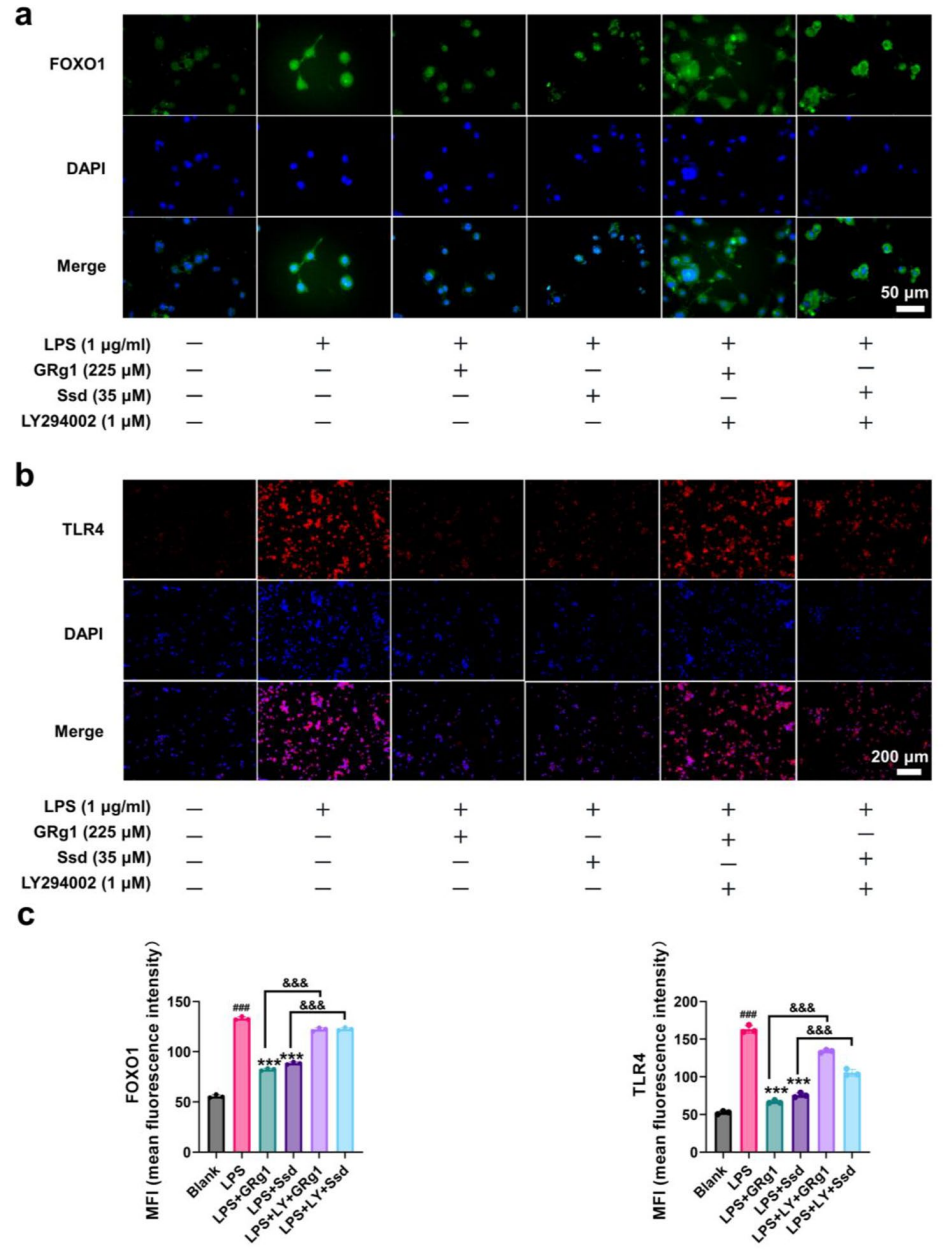
Immunocytofluorescent staining of BV2 cells. (**a**) The image of FOXO1 nucleus translocation was viewed with a confocal scanning microscope (*n* = 3; Scale bar: 50 μm). (**b**) The image of TLR4 was viewed with a confocal scanning microscope (*n* = 3; Scale bar: 200 μm). (**c**) The mean fluorescence intensity (MFI) of FOXO1 and TLR4 was analyzed. (n = 3). One-way ANOVA: # *P* < 0.05, ## *P* < 0.01, ### *P* < 0.001 vs. blank group; * *P* < 0.05, ** *P* < 0.01, *** *P* < 0.001 vs. LPS group. & *P* < 0.05, && *P* < 0.01, &&& *P* < 0.001 vs. LPS + LY + GRg1 group or LPS + LY + Ssd group

To investigate the role of microglial cells in the progression of brain diseases and brain aging, BV2 microglial cells were utilized for in vitro experiments [58, 59]. As depicted in Fig. 7a, stimulation with LPS led to a significant increase in the expressions of TNF-α (*P* < 0.001, F = 0.5273), IL-1β (*P* < 0.001, F = 0.3710), and IL-6 (*P* < 0.001, F = 0.6855) in BV2 cells compared with the blank group, indicating the promotion of inflammation. Treatment with the main compounds of KJG (GRg1 and Ssd) resulted in a reduction in the levels of TNF-α (*P* < 0.001), IL-1β (*P* < 0.001), and IL-6 (*P* < 0.001) in BV2 cells pre-treated with LPS, while this trend was blocked by LY294002, indicating the involvement of the PI3K/AKT/FOXO1 signaling pathway. Furthermore, the protein levels of p-AKT (*P* < 0.01, F = 1.426), p-PI3K (*P* < 0.001, F = 0.6014), and p-FOXO1 (*P* < 0.001, F = 0.5982) were decreased by LPS and LY294002 compared to the blank group, whereas TLR4 (*P* < 0.001, F = 0.2733) expression was upregulated (Fig. 7b). However, the harmful effects of the above trends were mitigated by GRg1 and Ssd via suppression of TLR4 activation in BV2 microglial cells induced by LPS (*P* < 0.05). The binding of multiple transcriptional elements in TLR4 protein is a characteristic of FOXO1, which can shuttle nuclear/cytoplasmic by phosphorylation [55, 60]. Moreover, the Nuclear Protein Extraction Kit (R0050, Solarbio) was used to prepare the cytoplasmic and nuclear fractions, and the WB results demonstrated that treatment with GRg1 and Ssd increased the cytoplasmic p-FOXO1 protein level, resulting in FOXO1 nuclear exclusion and degradation (Fig. 7c). The immunofluorescence microscopy results (Fig. 8a) showed that GRg1 and Ssd enhanced the phosphorylation of FOXO1, which induced its nuclear exclusion and downregulated its transcriptional activity in LPS-stimulated BV2 cells. In contrast, the expression of TLR4 was downregulated (Fig. 8b). LY294002 treatment, on the other hand, decreased the p-FOXO1 level and augmented the FOXO1 expression, which resulted in the reversal of the above changes (Fig. 8c). Overall, these findings indicated that GRg1 and Ssd, the main compounds of KJG, could reduce the neuroinflammation response in BV2 microglial cells by regulating the expression of TLR4 induced by LPS through the PI3K/AKT/FOXO1 signaling pathway.

## Discussion

KJG is a well-known TCM prescription for the treatment of depression and has shown significant beneficial effects in TCM clinical practice [13–16]. Our previous animal experiments have demonstrated that KJG can improve the depression-like behavior of vascular depression rats by regulating GFAP levels in the hippocampus [61]. However, the precise mechanisms underlying its therapeutic effects on neuroinflammation are not well understood. A series of network pharmacology and molecular docking analysis suggested that the main compounds of KJG (GRg1 and Ssd) could regulate the expression of TLR4 and PI3K/AKT/FOXO1-related proteins, suggesting that the anti-depressant effects of KJG are likely related to the remission of inflammation through the elimination of TLR4 and the subsequent activation of the PI3K/AKT/FOXO1 pathway. To verify the assumption, we performed a series of experiments. First, a combination of chronic and acute stress-induced animal models was conducted to support the hypothesis. Second, the concentrations of TNF-α, IL-1β, and IL-6 and expression of PI3K/AKT/FOXO1 related protein were detected in vivo animal assy. Furthermore, the above tests were also conducted in vitro cellular experiments.

The study found that KJG decreased the production of pro-inflammatory factors (TNF-α, IL-1β, and IL-6) in serum and TLR4 in hippocampal tissue, coinciding with ameliorating depressive behaviors. However, further investigation is needed to confirm the involvement of TLR4 in this process. The TLR4 pathway has been reported to serve as the primary mediator leading to depressive behaviors [62–64]. In the present study, a high level of TLR4 resulted in a loss of neural cells, increased concentrations of TNF-α, IL-1β, and IL-6, and more severe symptoms of behavioral abnormalities in depressive mice. As expected, the injection of TAK242 reversed the deepening of inflammation and depression-like behaviors. Notably, KJG exhibited significant positive treatment effects, including reduced TLR4 expression triggered by LPS, decreased levels of inflammatory cytokines, and amelioration of depressive behaviors, which was consistent with the results of TAK242. Therefore, it can be confirmed that TLR4 is involved in the process of depression, and KJG may be used as an effective anti-depressant medication.

**Fig. 9:**
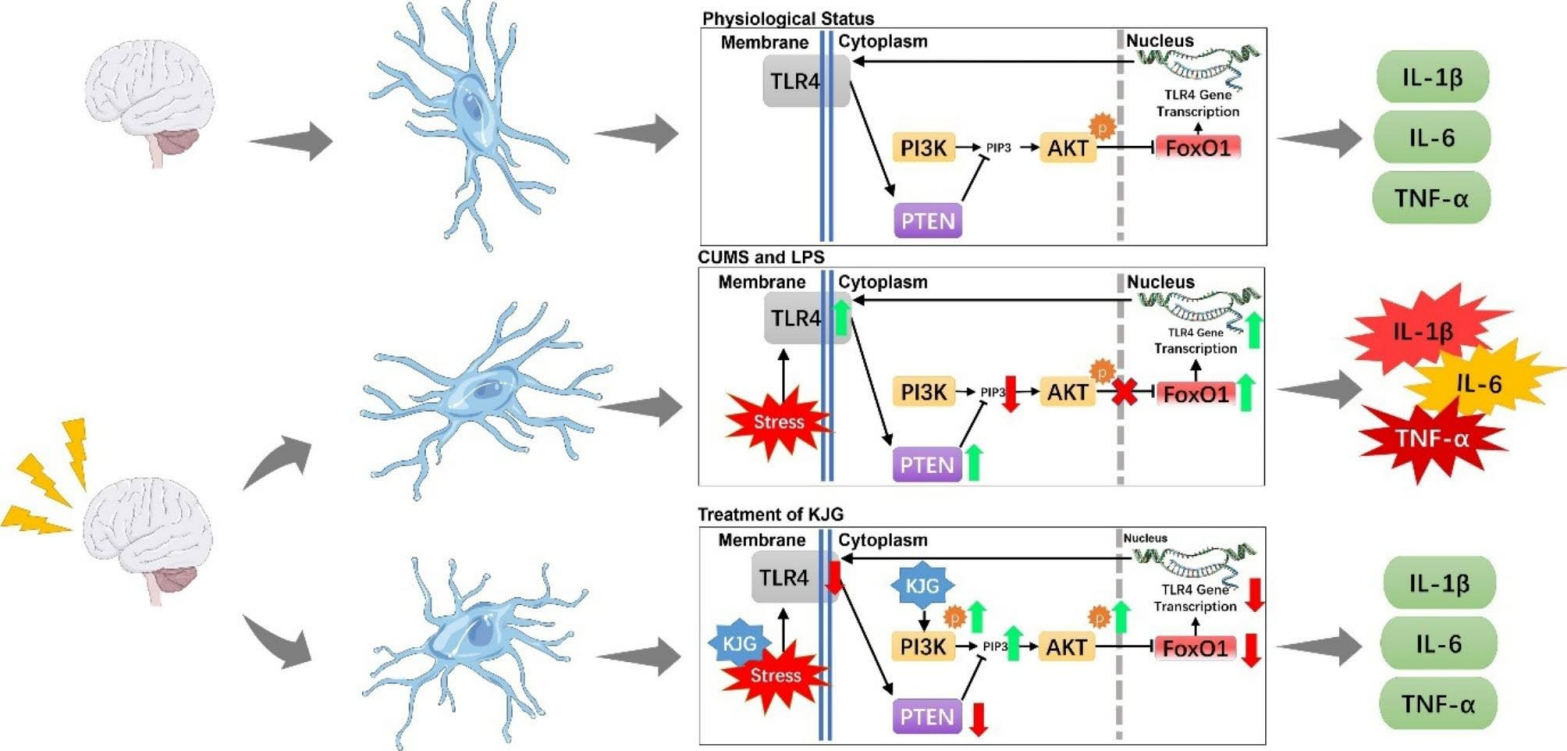
Diagram of the proposed molecular mechanism of the anti-depressive effect of KJG. In physiological status, TLR4 and PI3K/AKT signaling pathways are inactivated, and the increased secretion of pro-inflammatory factors (TNF-α, IL-6, IL-1β) is not observed. After CUMS and LPS exposure, the TLR4 pathway is triggered, followed by the PTEN-induced inhibition of the PI3K/AKT signaling pathway and the promoted transcriptional activity of FOXO1, as well as the increased secretion of pro-inflammatory factors. Conversely, the KJG-mediated anti-depressant effect can decrease TLR4 but increase PI3K/AKT expression levels, inhibiting FOXO1 activity by promoting nuclear exportation and reducing pro-inflammatory secretion factors

To investigate the involvement of the PI3K/AKT pathway in depression, we utilized LY294002 to inhibit the activation of PI3K. Our results showed that the robust protective effect of KJG against depression was abolished by LY294002, indicating that the anti-depressant effect of KJG is likely associated with the PI3K/AKT pathway. Conversely, removing the PI3K/AKT pathway inhibitor improved the neuroinflammatory status induced by KJG, as evidenced by decreased levels of TLR4, increased levels of p-PI3K and p-AKT, and repressed activity of FOXO. In addition, PTEN, which is correlated with neuronal development by controlling synaptic plasticity and neuronal excitability in the adult central nervous system [65–67], was found to significantly modulate the feedback mechanism through the PI3K/AKT pathway in depression. Our study suggests that KJG may achieve its anti-depressant effect by downregulating TLR4 expression via activation of the PI3K/AKT/FOXO1 pathway (Fig. 9).

Apart from this, the study observed the mechanisms of the main compounds of KJG (GRg1 and Ssd) for treating depression in LPS-stimulated microglial BV2 cells. The results showed that GRg1 and Ssd could effectively inhibit the production of TLR4 and FOXO1 while enhancing the sensitivity of the PI3K/AKT pathway. The results in vitro may explain that the KJG-mediated anti-depressant effect was attributed to the inhibition of FOXO1-mediated transcription by promoting its nuclear export.

However, this study has some limitations. The molecular targets of KJG may not act solely via the PI3K/AKT/FOXO1 signaling pathway, and the possibility of other mechanisms cannot be excluded. In addition, the other effective compounds or ingredients in KJG with anti-depressant activity remained unclear. Therefore, further studies will be required to explore the mechanism of action of KJG in more detail.

## Conclusion

In summary, we utilized network pharmacology and molecular docking approaches to identify the active compounds and signaling pathways associated with KJG in treating depression. Through bioinformatics analysis, GRg1 and Ssd were identified as the principal active ingredients in KJG. Further investigation demonstrated that KJG exerts its anti-depressant effects by regulating the expression of TLR4, FOXO1, and PTEN via the PI3K/AKT signaling pathway. In vitro and in vivo experiments confirmed that KJG is an effective treatment for depression by downregulating TLR4 expression to reduce neuroinflammation through the PI3K/AKT/FOXO1 pathway. This study lays a foundation for comprehensively exploring the pathogenic mechanisms of KJG. Taken together, these findings highlight the potential of systems pharmacology-based network analyses for investigating the anti-depressant mechanisms of TCMs.

## Electronic supplementary material

Below is the link to the electronic supplementary material.

**Additional file 1:** Western blotting results of BV2 cells.

**Additional file 2:** Western blotting results of Animals.

**Additional file 3:** Supplementary Figures and Tables.

## Data Availability

The datasets used and analyzed during the present study are available from the corresponding author upon reasonable request.

## Abbreviations

AKT1: AKT serine/threonine kinase 1
CUMS: Chronic unpredictable mild stress
DL: Drug similarity
FOXO1: Forkhead box protein O1
FST: Forced swimming test
GRg1: ginsenoside Rg1
IL-1β: Interleukin-1β
IL-6: Interleukin-6
KJG: Kaixin Jieyu Granule
LPS: Lipopolysaccharide
OB: Oral bioavailability
OFT: Open field test
PI3K: phosphatidylinositide 3-kinases
SPT: Sucrose preference test
Ssd: Saikosaponin d
TCM: Traditional Chinese medicine
TLR4: Toll-like receptor 4
TNF-α: Tumor necrosis factor-α
TST: Tail suspension test
