# Supplementary material for: Kaixin Jieyu Granule attenuates neuroinflammation-induced depressive-like behavior through TLR4/PI3K/AKT/FOXO1 pathway: a study of network pharmacology and experimental validation
Source: BMC Complement Med Ther. 2023 May 12;23:156. doi: 10.1186/s12906-023-03970-5 (PMC10182664; doi:10.1186/s12906-023-03970-5)
Supplement: Supplementary file 1 — Additional file 1: Western blotting results of BV2 cells. [file 12906_2023_3970_MOESM1_ESM.pdf]

Western blotting results of BV2 cells

The selected protein bands of the manuscript were highlighted yellow in the raw data below.

Samples number	Groups
①	Blank
②	LPS
③	LPS+GRg1
④	LPS+Ssd
⑤	LPS+LY+GRg1
⑥	LPS+LY+Ssd

β-actin

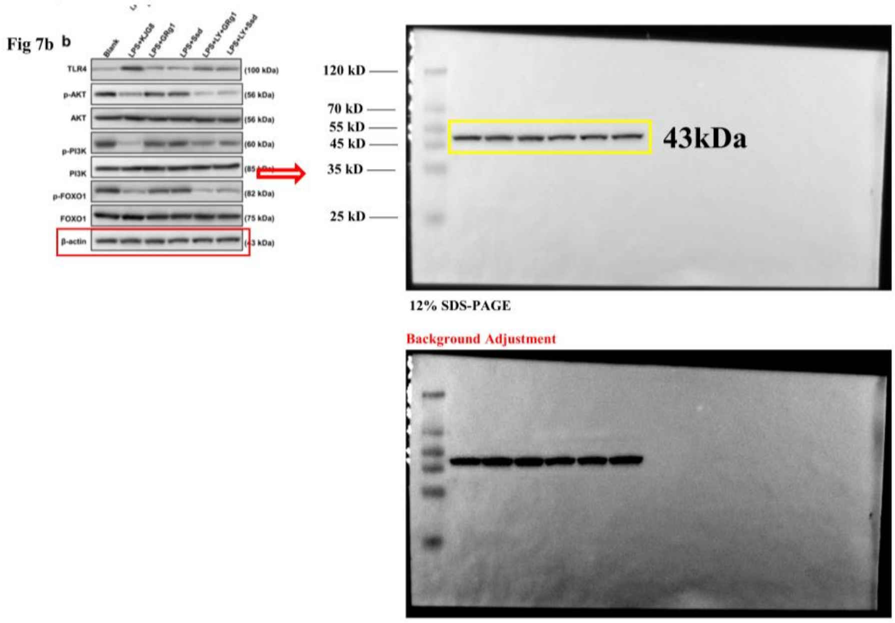

Yellow: ①, ②, ③, ④, ⑤, ⑥ represent western blot analysis shown in **Fig 7b.**

The order of loading for western blotting was as follows:

①, ②, ③, ④, ⑤, ⑥

FoxO1

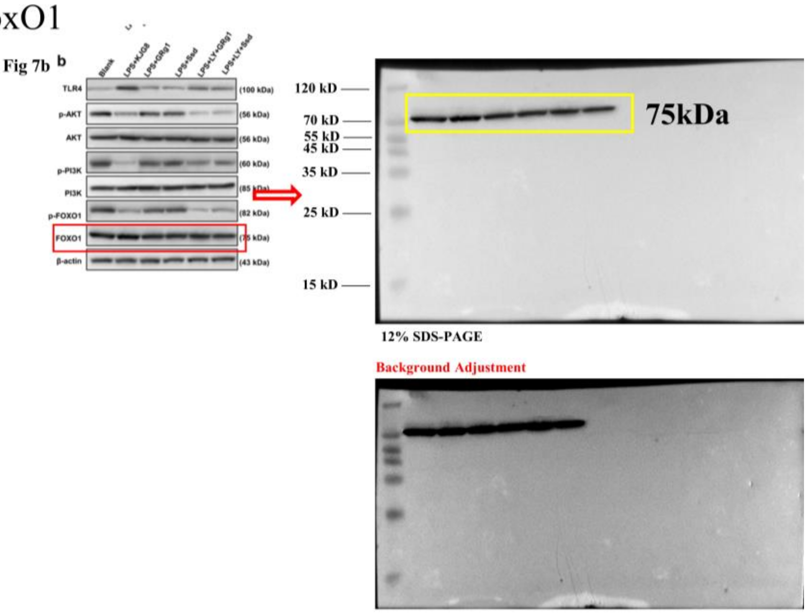

Yellow: ①, ②, ③, ④, ⑤, ⑥ represent western blot analysis shown in **Fig 7b**.

The order of loading for western blotting was as follows:

①, ②, ③, ④, ⑤, ⑥

p-FoxO1

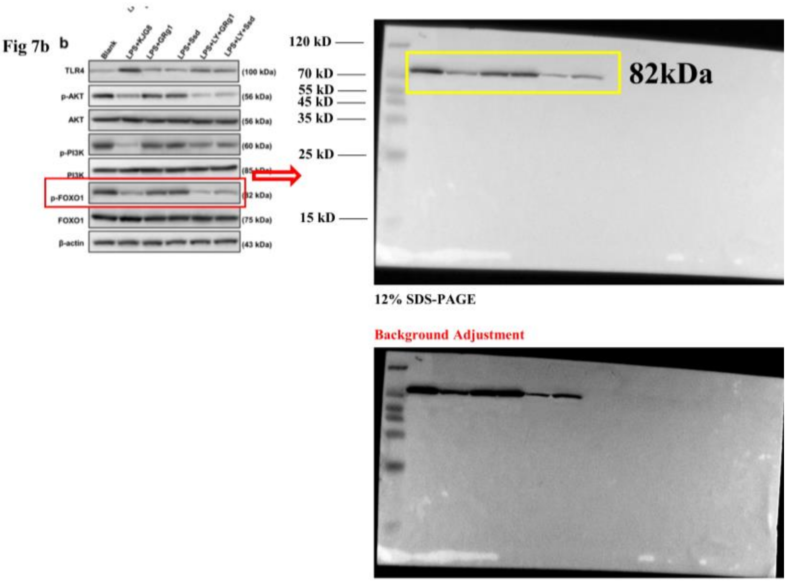

Yellow: ①, ②, ③, ④, ⑤, ⑥ represent western blot analysis shown in **Fig 7b**.

The order of loading for western blotting was as follows:

①, ②, ③, ④, ⑤, ⑥

PI3K

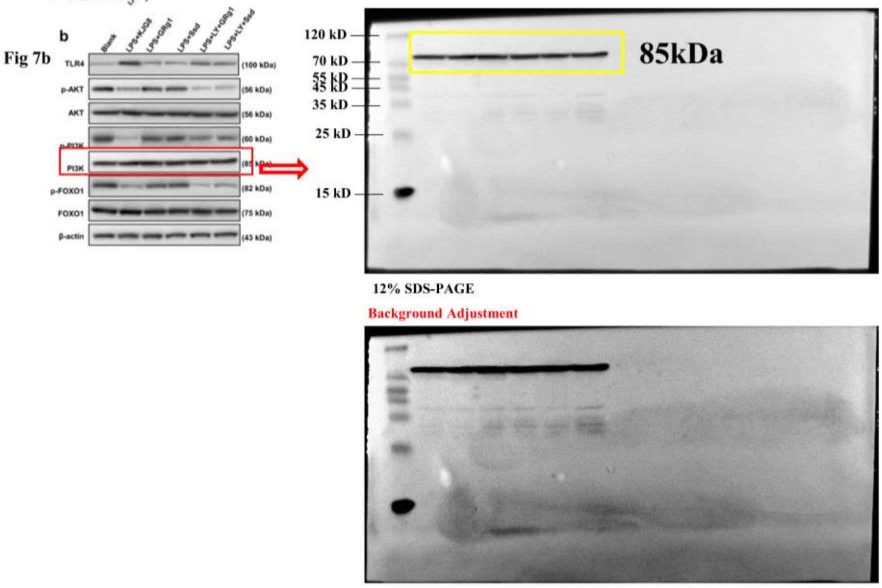

Yellow: ①, ②, ③, ④, ⑤, ⑥ represent western blot analysis shown in **Fig 7b**.

The order of loading for western blotting was as follows:

①, ②, ③, ④, ⑤, ⑥

p-PI3K

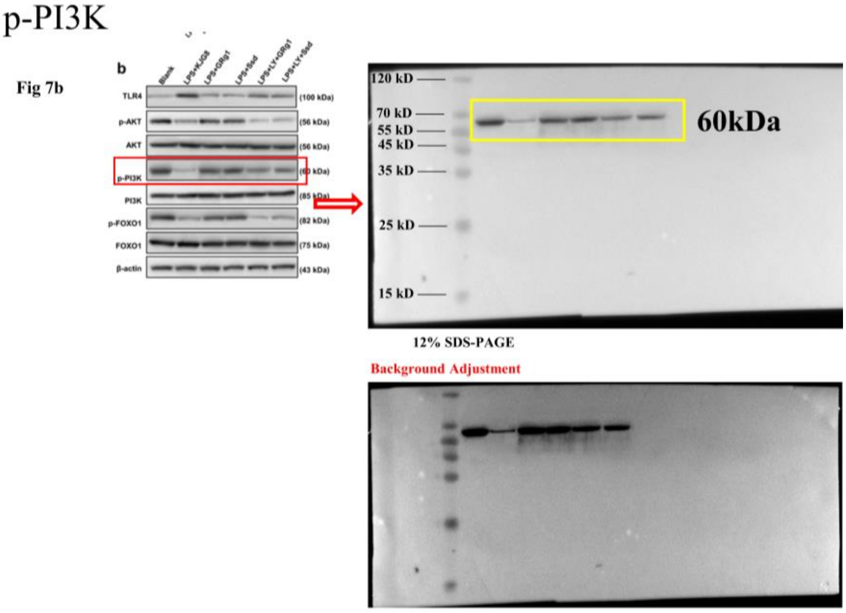

Yellow: ①, ②, ③, ④, ⑤, ⑥ represent western blot analysis shown in

Fig 7b.

The order of loading for western blotting was as follows:

①, ②, ③, ④, ⑤, ⑥

Akt

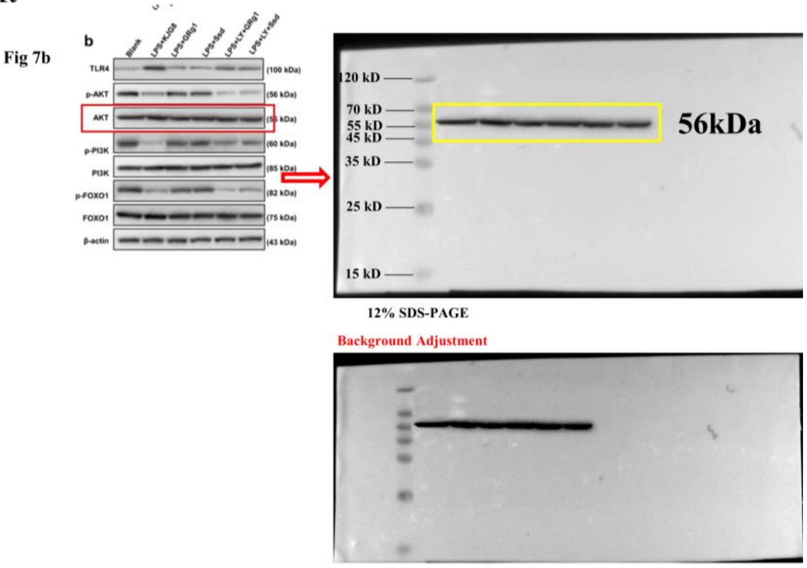

Yellow: ①, ②, ③, ④, ⑤, ⑥ represent western blot analysis shown in

Fig 7b.

The order of loading for western blotting was as follows:

①, ②, ③, ④, ⑤, ⑥

p-Akt

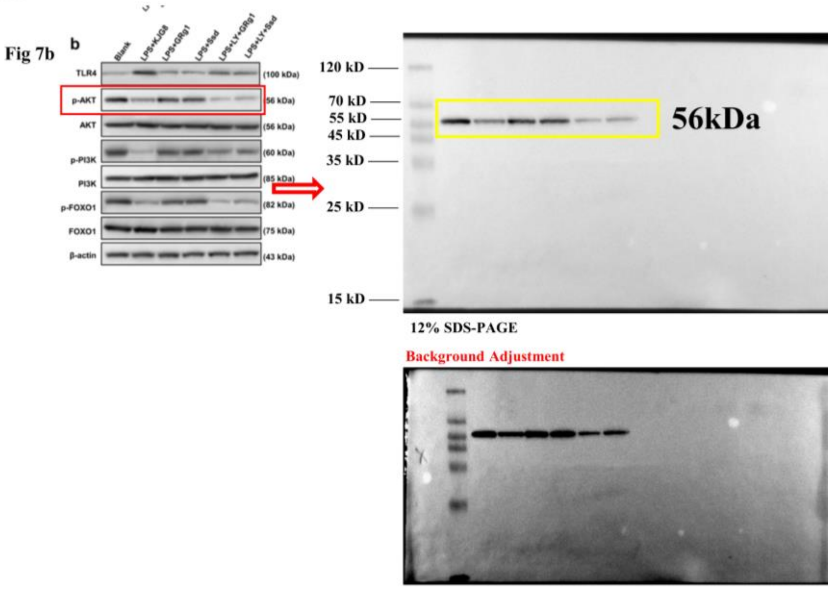

Yellow: ①, ②, ③, ④, ⑤, ⑥ represent western blot analysis shown in

Fig 7b.

The order of loading for western blotting was as follows:

①, ②, ③, ④, ⑤, ⑥

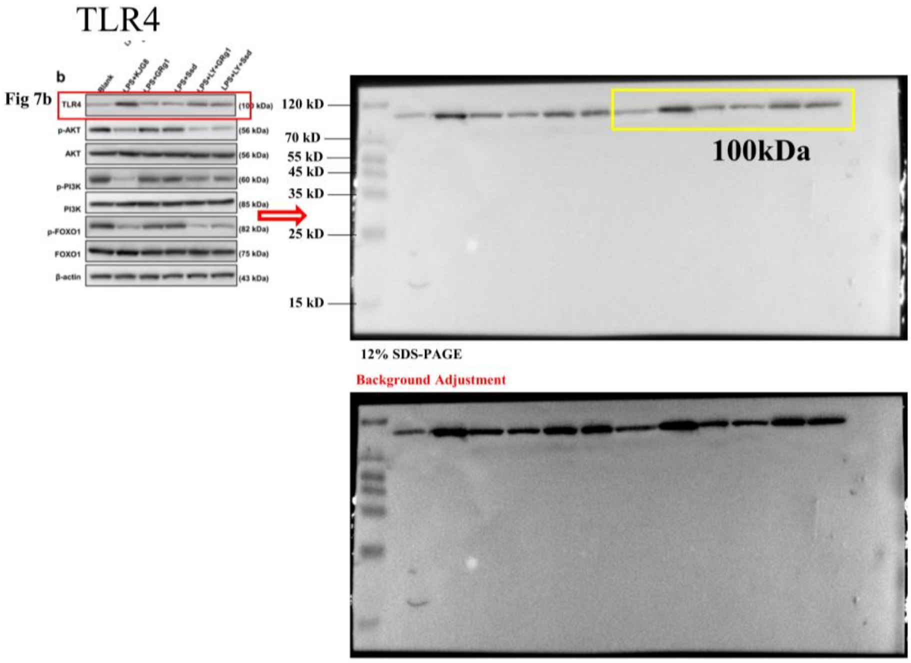

Yellow: ①, ②, ③, ④, ⑤, ⑥ represent western blot analysis shown in

Fig 7b.

The order of loading for western blotting was as follows:

①, ②, ③, ④, ⑤, ⑥, ①, ②, ③, ④, ⑤, ⑥

β-actin

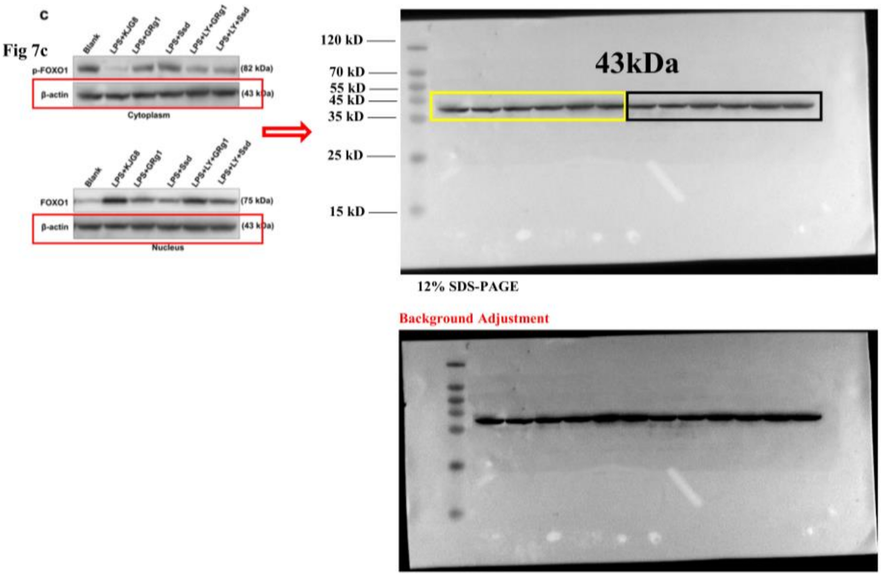

Yellow: ①, ②, ③, ④, ⑤, ⑥ represent western blot analysis shown in **Fig 7c**, cytoplasm;

Black: ①, ②, ③, ④, ⑤, ⑥ represent western blot analysis shown in **Fig 7c**, nucleus.

The order of loading for western blotting was as follows:

①, ②, ③, ④, ⑤, ⑥

FoxO1

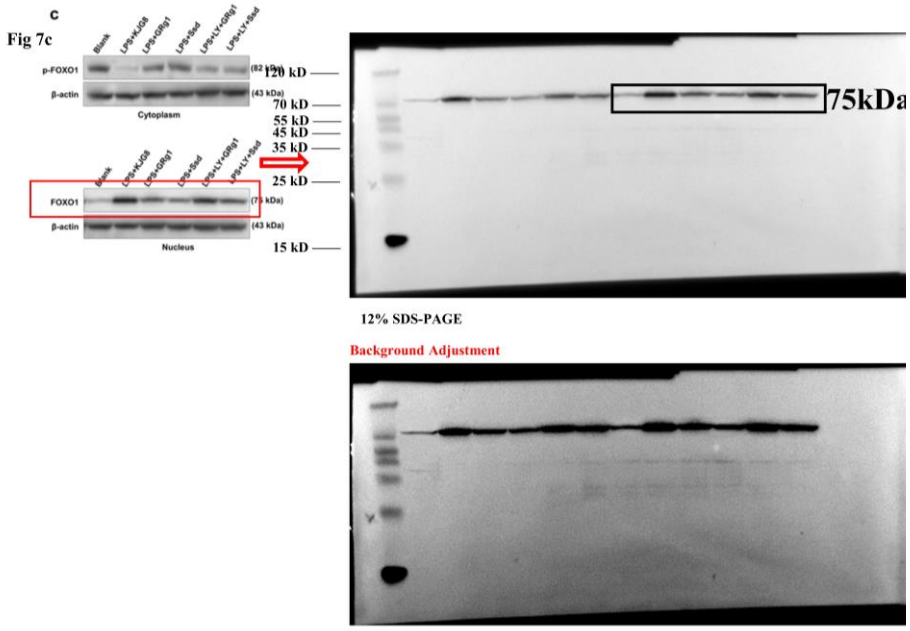

Black: ①, ②, ③, ④, ⑤, ⑥ represent western blot analysis shown in **Fig 7c**, nucleus.

The order of loading for western blotting was as follows:

①, ②, ③, ④, ⑤, ⑥

p-FoxO1

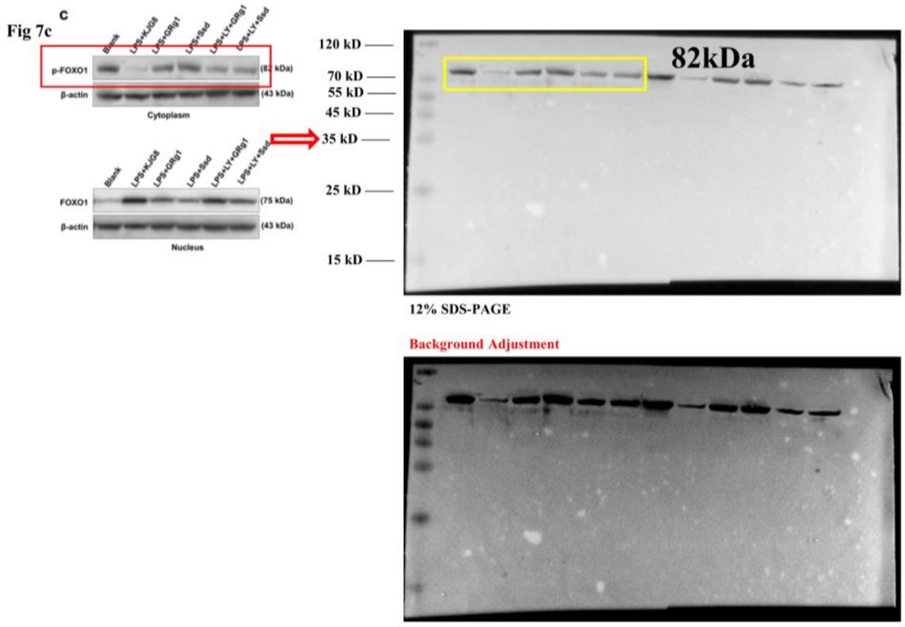

Yellow: ①, ②, ③, ④, ⑤, ⑥ represent western blot analysis shown in

Fig 7c, cytoplasm.

The order of loading for western blotting was as follows:

①, ②, ③, ④, ⑤, ⑥
