# Supplementary material for: Kaixin Jieyu Granule attenuates neuroinflammation-induced depressive-like behavior through TLR4/PI3K/AKT/FOXO1 pathway: a study of network pharmacology and experimental validation
Source: BMC Complement Med Ther. 2023 May 12;23:156. doi: 10.1186/s12906-023-03970-5 (PMC10182664; doi:10.1186/s12906-023-03970-5)
Supplement: Supplementary file 2 — Additional file 2: Western blotting results of Animals. [file 12906_2023_3970_MOESM2_ESM.pdf]

Western blotting results of Animals

The selected protein bands of the manuscript were highlighted yellow in the raw data below.

Samples number	Groups
①	Control
②	control+KJG8(8000 mg crude drug/kg/d)
③	CUMS
④	CUMS+FLU(3.33mg/kg/d)
⑤	CUMS+KJG4(4000 mg crude drug/kg/d)
⑥	CUMS+KJG8 (8000 mg crude drug/kg/d)
⑦	CUMS +LY294002+KJG8(8000 mg crude drug/kg/d)
⑧	LPS(0.83 mg/kg/d)
⑨	LPS+TAK242（3 mg/kg/d）
⑩	LPS+ KJG8(8000 mg crude drug/kg/d)

The original images mentioned in the manuscript:

β-actin

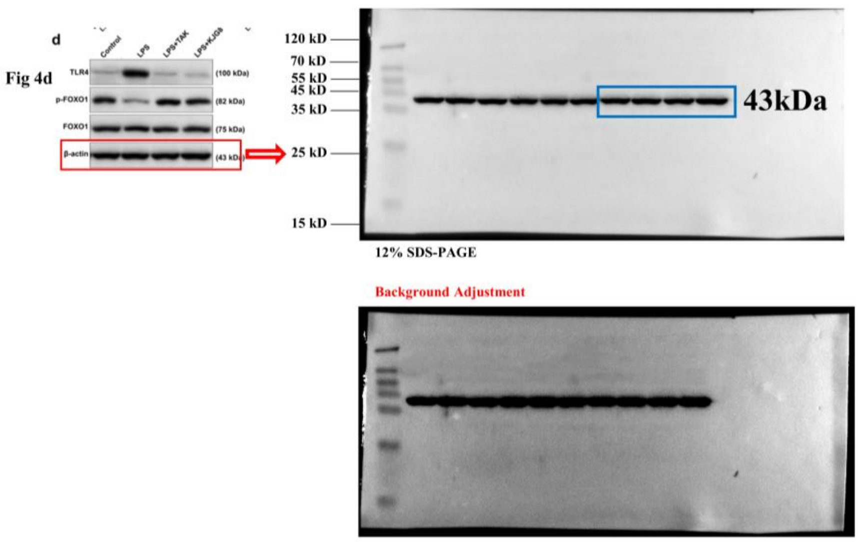

Blue: ①, ⑧, ⑨, ⑩ represent western blot analysis shown in **Fig 4d.**

The order of loading for western blotting was as follows:

①, ②, ③, ④, ⑤, ⑥, ①, ⑧, ⑨, ⑩

FoxO1

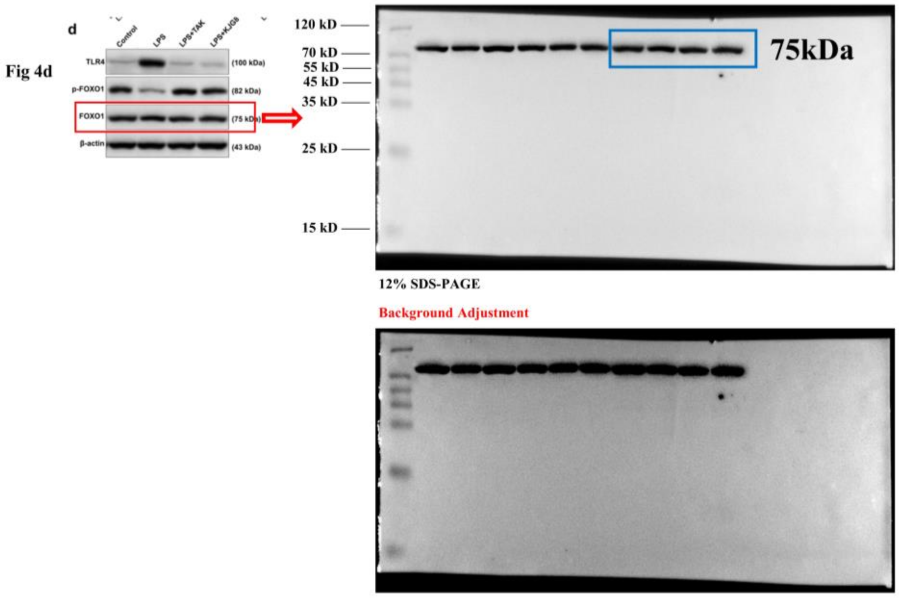

Blue: ①, ⑧, ⑨, ⑩ represent western blot analysis shown in **Fig 4d.**

The order of loading for western blotting was as follows:

①, ②, ③, ④, ⑤, ⑥, ①, ⑧, ⑨, ⑩

p-FoxO1

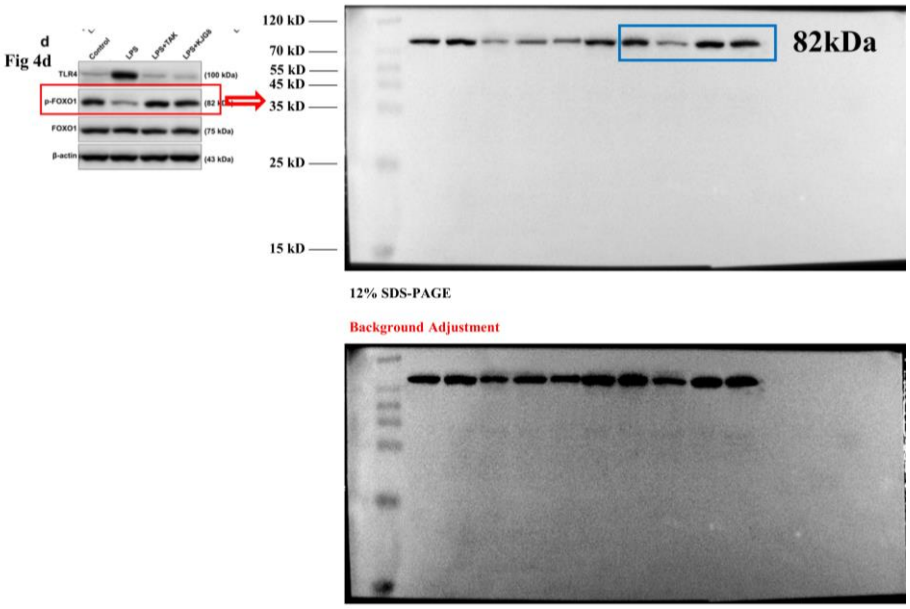

Blue: ①, ⑧, ⑨, ⑩ represent western blot analysis shown in **Fig 4d.**

The order of loading for western blotting was as follows:

①, ②, ③, ④, ⑤, ⑥, ①, ⑧, ⑨, ⑩

TLR4

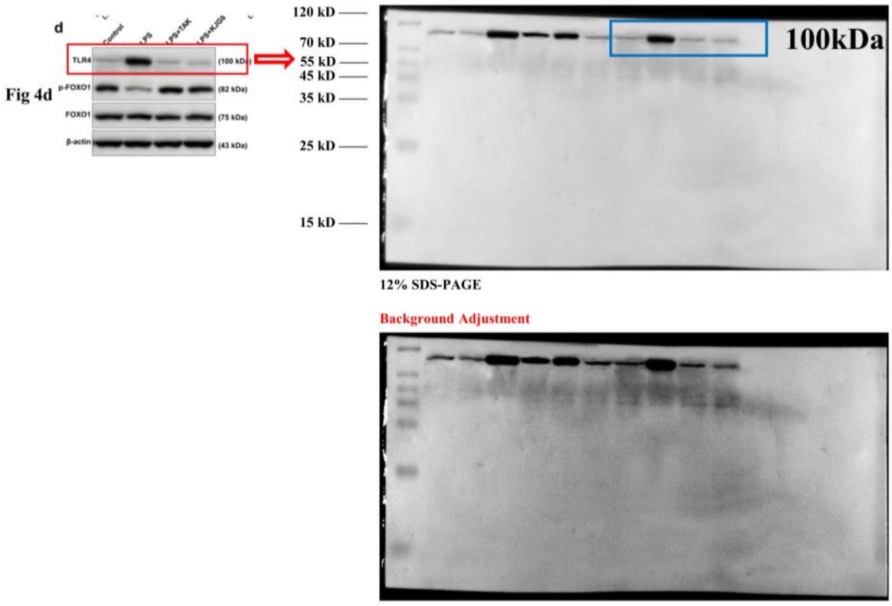

Blue: ①, ⑧, ⑨, ⑩ represent western blot analysis shown in **Fig 4d.**

The order of loading for western blotting was as follows:

①, ②, ③, ④, ⑤, ⑥, ①, ⑧, ⑨, ⑩

β-actin

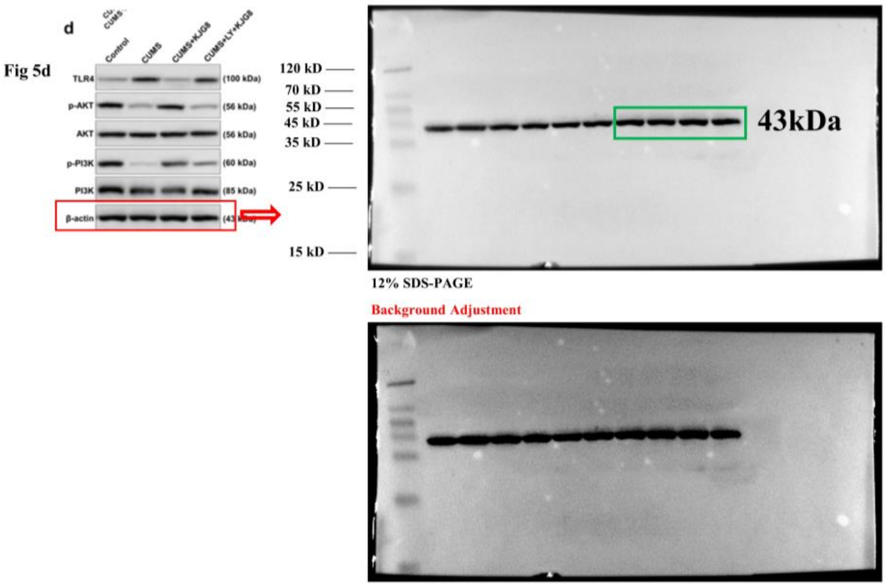

Green: ①, ③, ⑥, ⑦ represent western blot analysis shown in **Fig 5d**.

The order of loading for western blotting was as follows:

①, ②, ③, ④, ⑤, ⑥, ①, ③, ⑥, ⑦

PI3K

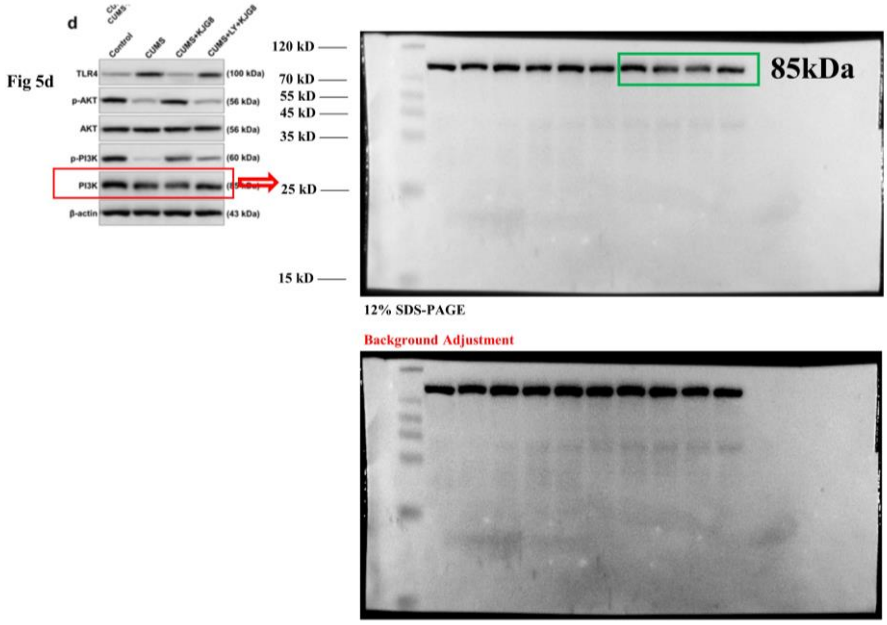

Green: ①, ③, ⑥, ⑦ represent western blot analysis shown in **Fig 5d**.

The order of loading for western blotting was as follows:

①, ②, ③, ④, ⑤, ⑥, ①, ③, ⑥, ⑦

p-PI3K

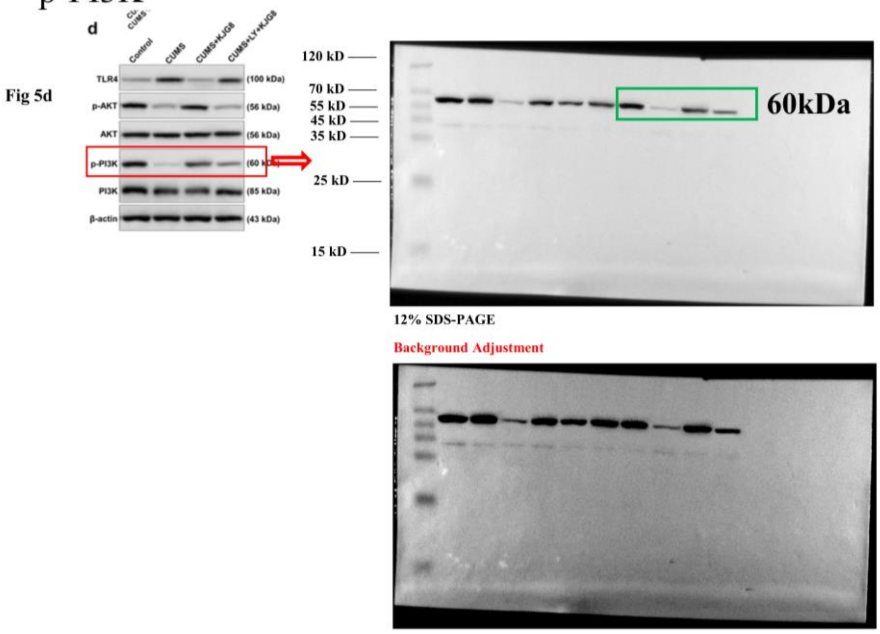

Green: ①, ③, ⑥, ⑦ represent western blot analysis shown in **Fig 5d.**

The order of loading for western blotting was as follows:

①, ②, ③, ④, ⑤, ⑥, ①, ③, ⑥, ⑦

Akt

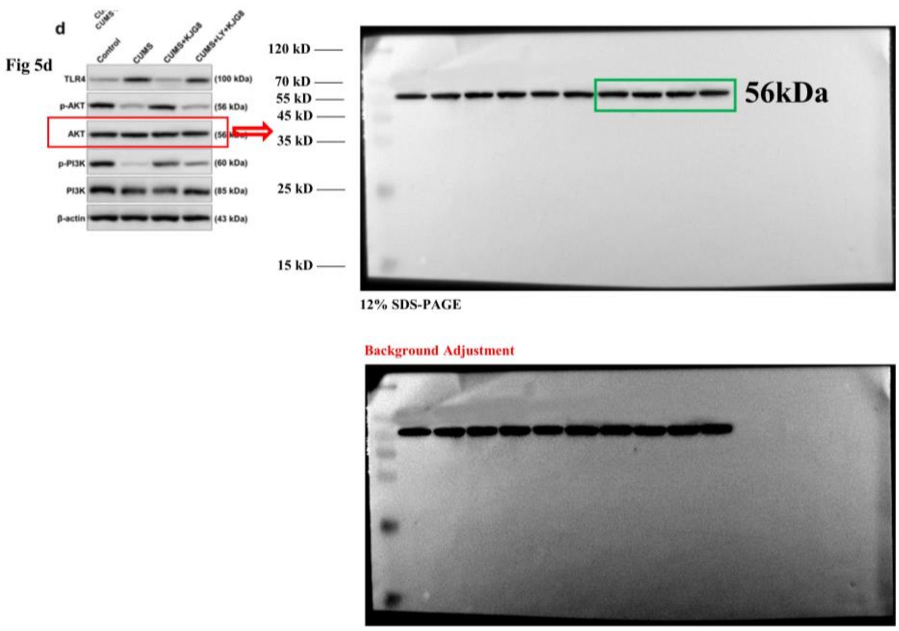

Green: ①, ③, ⑥, ⑦ represent western blot analysis shown in **Fig 5d.**

The order of loading for western blotting was as follows:

①, ②, ③, ④, ⑤, ⑥, ①, ③, ⑥, ⑦

p-Akt

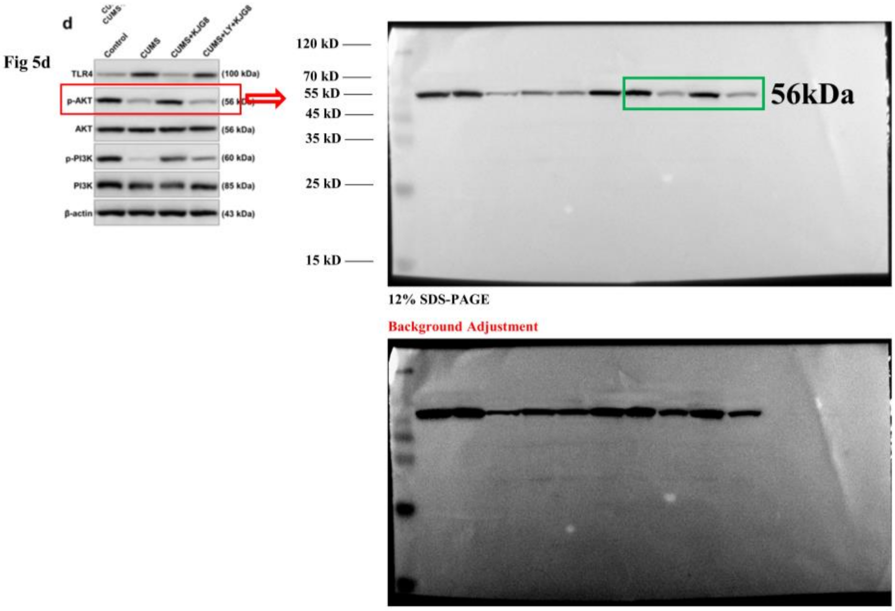

Green: ①, ③, ⑥, ⑦ represent western blot analysis shown in **Fig 5d**.

The order of loading for western blotting was as follows:

①, ②, ③, ④, ⑤, ⑥, ①, ③, ⑥, ⑦

TLR4

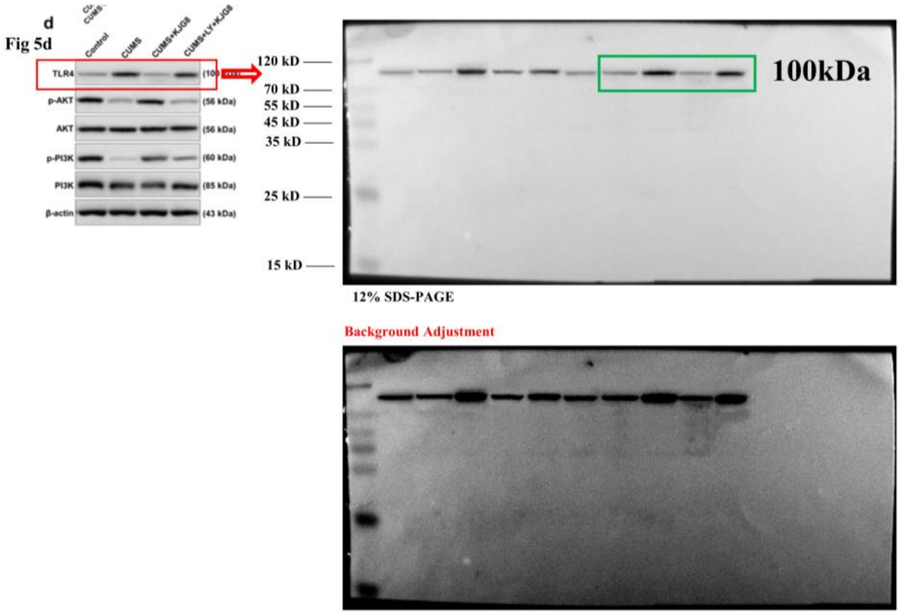

Green: ①, ③, ⑥, ⑦ represent western blot analysis shown in **Fig 5d**.

The order of loading for western blotting was as follows:

①, ②, ③, ④, ⑤, ⑥, ①, ③, ⑥, ⑦

β-actin

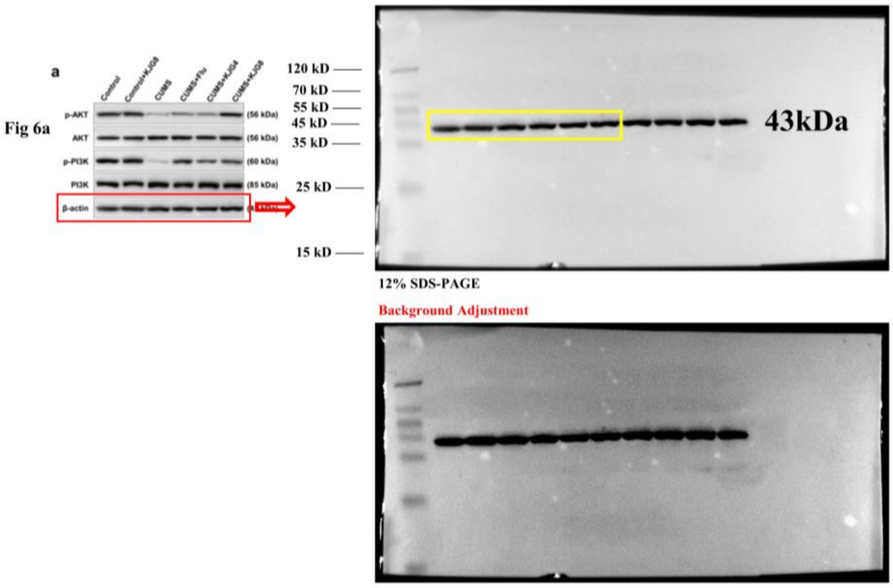

Yellow: ①, ②, ③, ④, ⑤, ⑥ represent western blot analysis shown in **Fig 6a**.

The order of loading for western blotting was as follows:

①, ②, ③, ④, ⑤, ⑥, ①, ③, ⑥, ⑦

PI3K

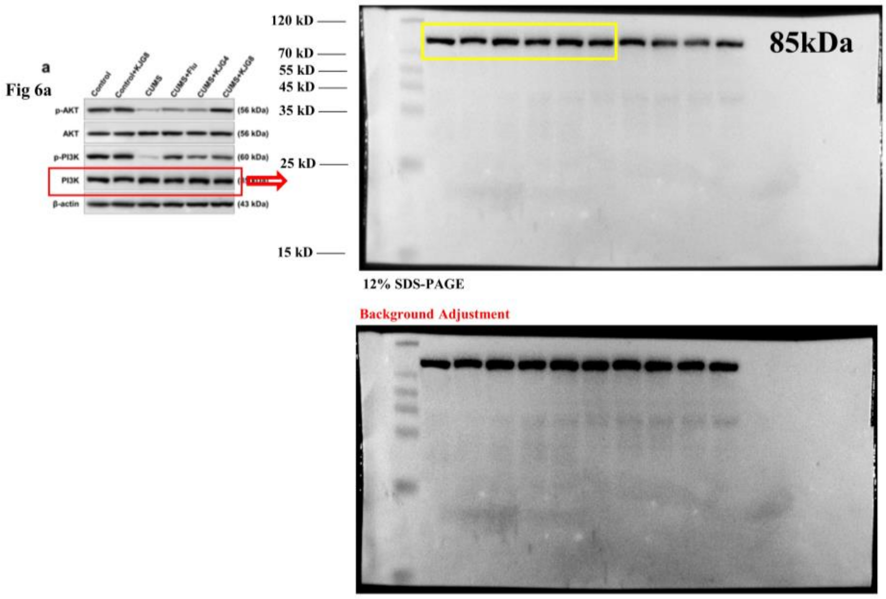

Yellow: ①, ②, ③, ④, ⑤, ⑥ represent western blot analysis shown in

Fig 6a.

The order of loading for western blotting was as follows:

①, ②, ③, ④, ⑤, ⑥, ①, ③, ⑥, ⑦

p-PI3K

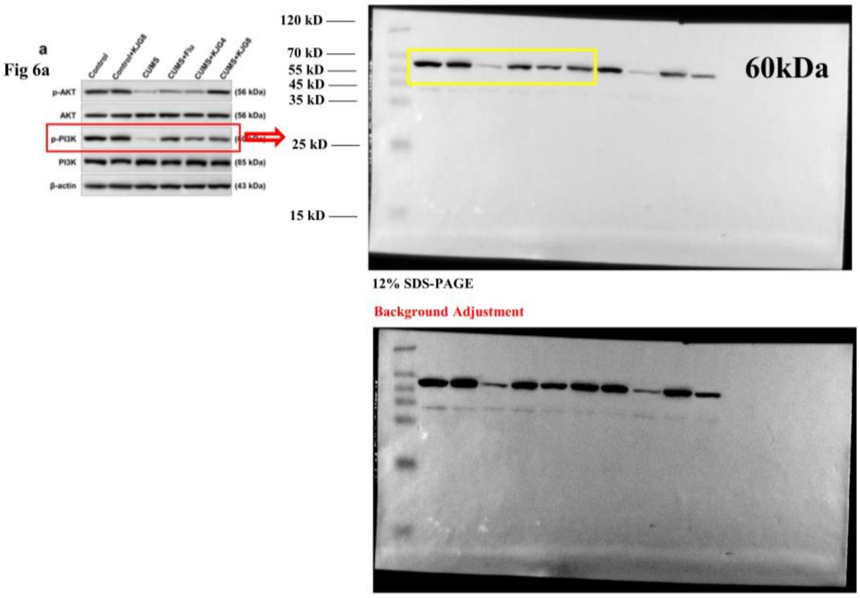

Yellow: ①, ②, ③, ④, ⑤, ⑥ represent western blot analysis shown in

Fig 6a.

The order of loading for western blotting was as follows:

①, ②, ③, ④, ⑤, ⑥, ①, ③, ⑥, ⑦

Akt

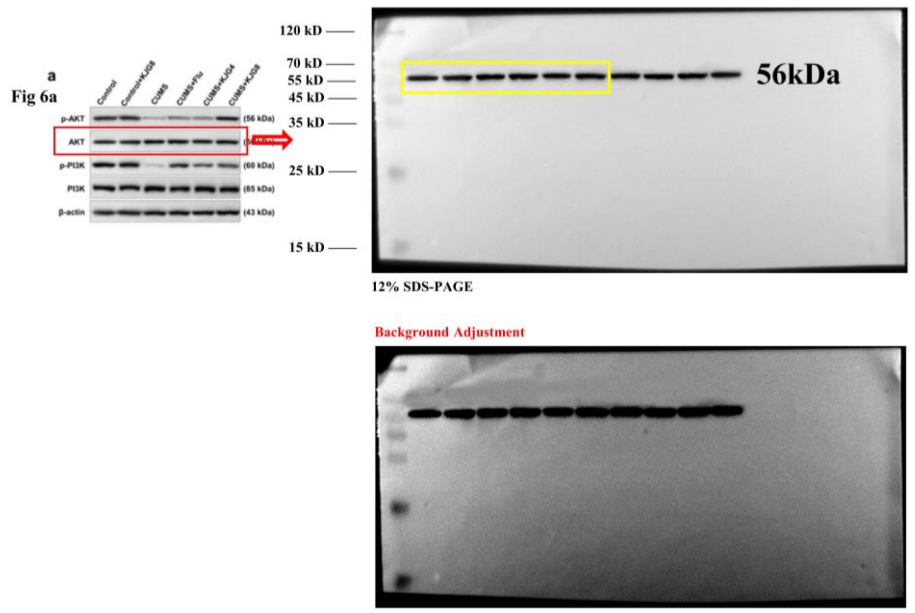

Yellow: ①, ②, ③, ④, ⑤, ⑥ represent western blot analysis shown in **Fig 6a**.

The order of loading for western blotting was as follows:

①, ②, ③, ④, ⑤, ⑥, ①, ③, ⑥, ⑦

p-Akt

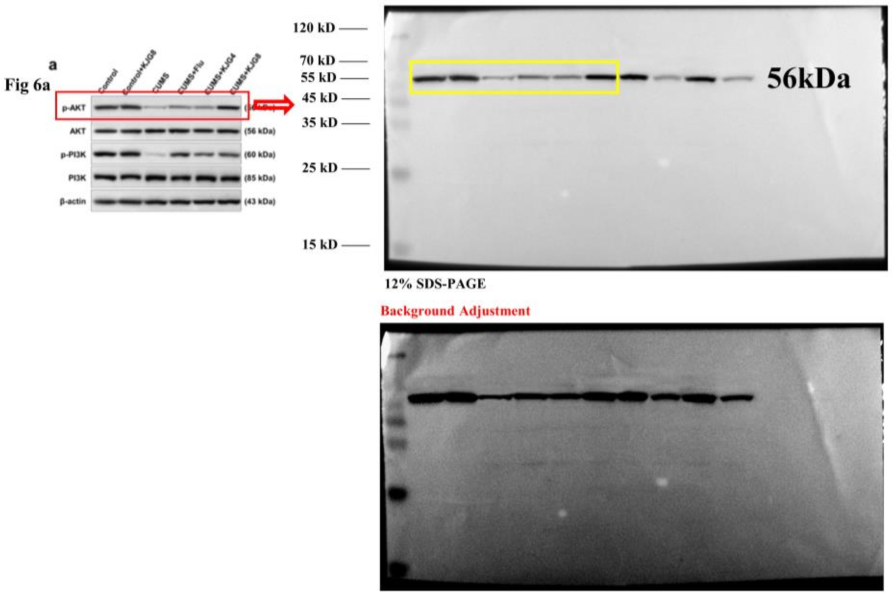

Yellow: ①, ②, ③, ④, ⑤, ⑥ represent western blot analysis shown in

Fig 6a.

The order of loading for western blotting was as follows:

①, ②, ③, ④, ⑤, ⑥, ①, ③, ⑥, ⑦

β-actin

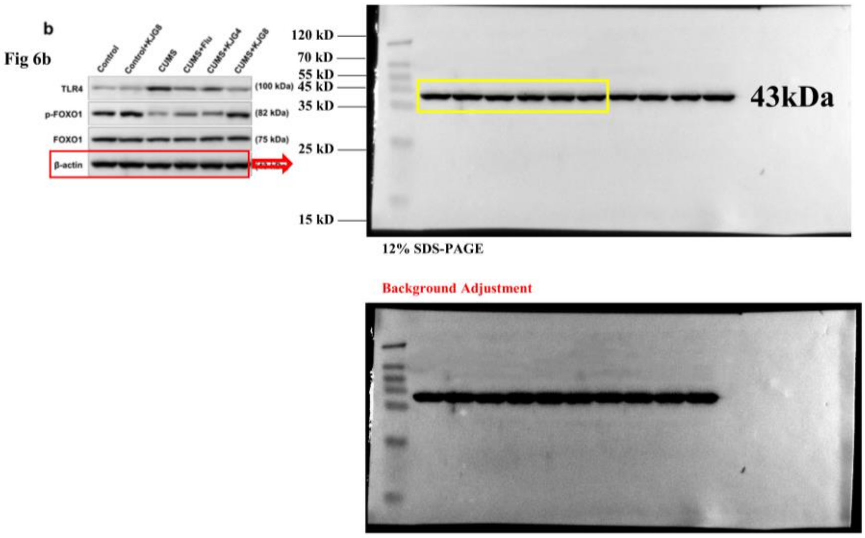

Yellow: ①, ②, ③, ④, ⑤, ⑥ represent western blot analysis shown in

Fig 6b.

The order of loading for western blotting was as follows:

①, ②, ③, ④, ⑤, ⑥, ①, ⑧, ⑨, ⑩

FoxO1

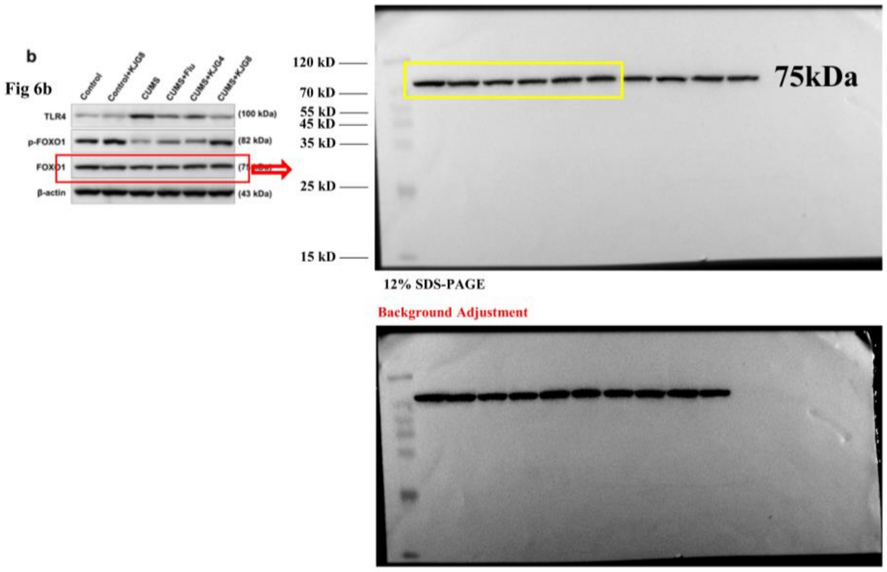

Yellow: ①, ②, ③, ④, ⑤, ⑥ represent western blot analysis shown in **Fig 6b**.

The order of loading for western blotting was as follows:

①, ②, ③, ④, ⑤, ⑥, ①, ⑧, ⑨, ⑩

p-FoxO1

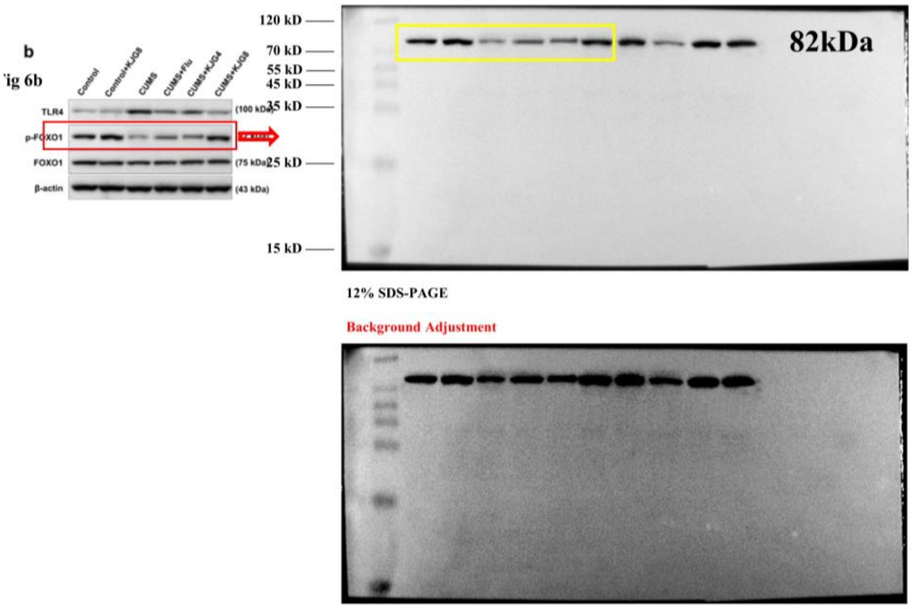

Yellow: ①, ②, ③, ④, ⑤, ⑥ represent western blot analysis shown in

Fig 6b.

The order of loading for western blotting was as follows:

①, ②, ③, ④, ⑤, ⑥, ①, ⑧, ⑨, ⑩

TLR4

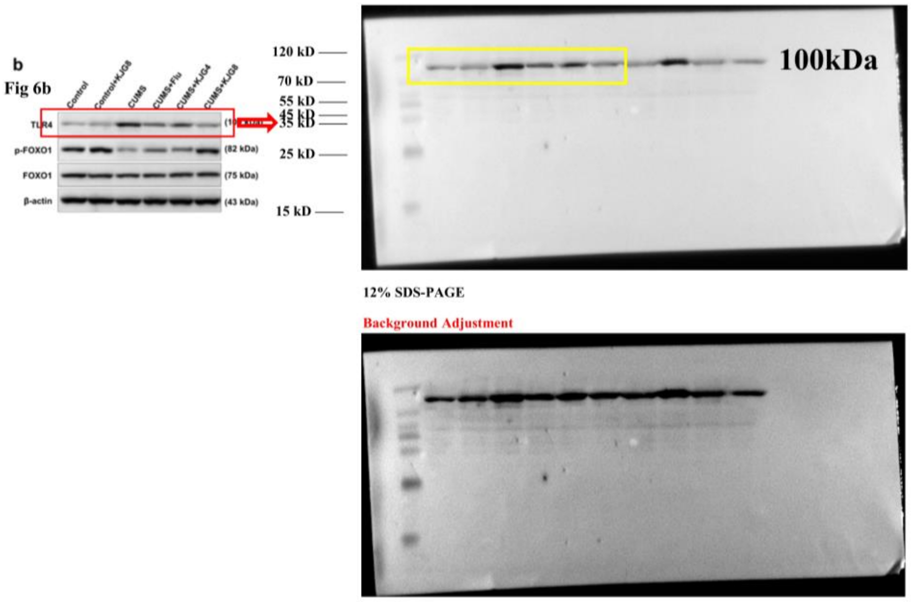

Yellow: ①, ②, ③, ④, ⑤, ⑥ represent western blot analysis shown in

Fig 6b.

The order of loading for western blotting was as follows:

①, ②, ③, ④, ⑤, ⑥, ①, ⑧, ⑨, ⑩

β-actin

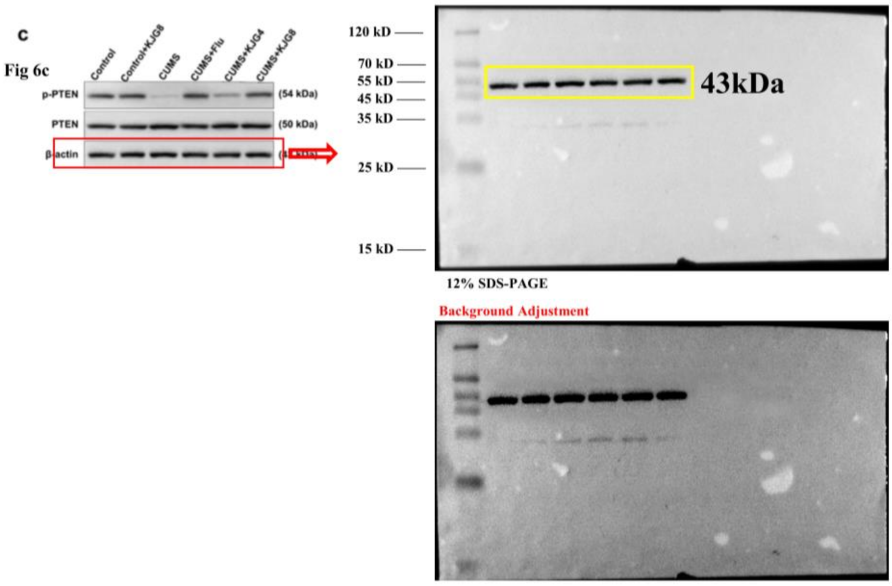

Yellow: ①, ②, ③, ④, ⑤, ⑥ represent western blot analysis shown in **Fig 6c**.

The order of loading for western blotting was as follows:

①, ②, ③, ④, ⑤, ⑥

PTEN

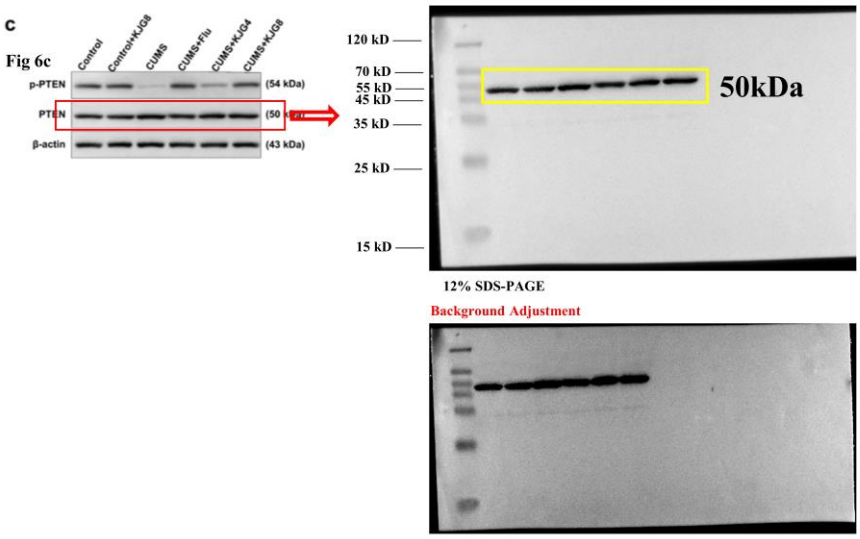

Yellow: ①, ②, ③, ④, ⑤, ⑥ represent western blot analysis shown in **Fig 6c**.

The order of loading for western blotting was as follows:

①, ②, ③, ④, ⑤, ⑥,

p-PTEN

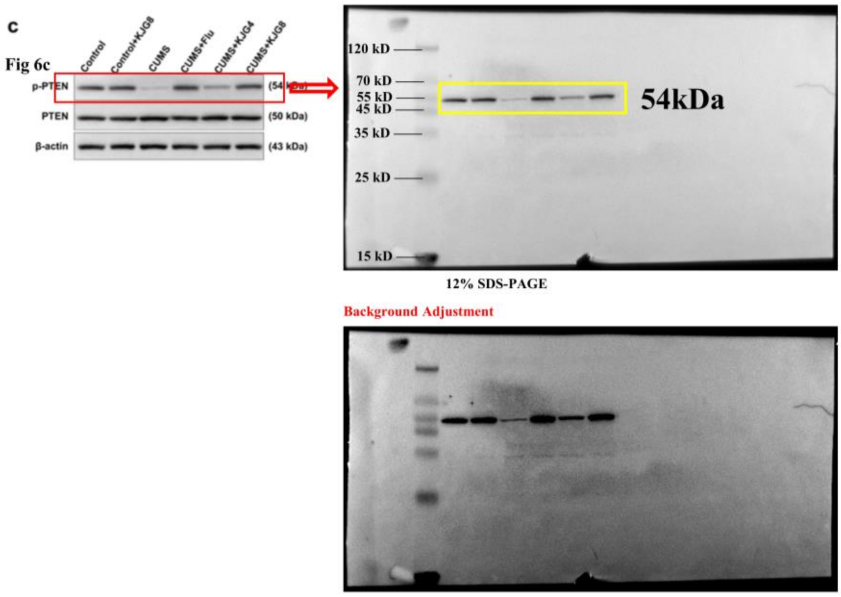

Yellow: ①, ②, ③, ④, ⑤, ⑥ represent western blot analysis shown in **Fig 6c**.

The order of loading for western blotting was as follows:

①, ②, ③, ④, ⑤, ⑥
