# Supplementary material for: Kaixin Jieyu Granule attenuates neuroinflammation-induced depressive-like behavior through TLR4/PI3K/AKT/FOXO1 pathway: a study of network pharmacology and experimental validation
Source: BMC Complement Med Ther. 2023 May 12;23:156. doi: 10.1186/s12906-023-03970-5 (PMC10182664; doi:10.1186/s12906-023-03970-5)
Supplement: Supplementary file 3 — Additional file 3: Supplementary Figures and Tables. [file 12906_2023_3970_MOESM3_ESM.docx]

**Supporting Information**

**Kaixin Jieyu Granule attenuates neuroinflammation-induced depressive-like behavior through TLR4/PI3K/AKT/FOXO1 pathway: a study of network pharmacology and experimental validation**

Manman Xu^1,2^^†^, Wujianwen Zhai^1†^, Ying Zhang^1^, Juhua Pan^1^, Jie Li^2^*, Shijing Huang^1^*

* Correspondence: [qfm2020jieli@yeah.net](mailto:qfm2020jieli@yeah.net), [hsjgam2878@163.com](mailto:hsjgam2878@163.com)

^1^Traditional Chinese Medicine Research and Development Center, Guang' Anmen Hospital, China Academy of Chinese Medical Sciences, Beijing 100053, China.

^2^Department of oncology, Guang' Anmen Hospital, China Academy of Chinese Medical Sciences, Beijing 100053, China.


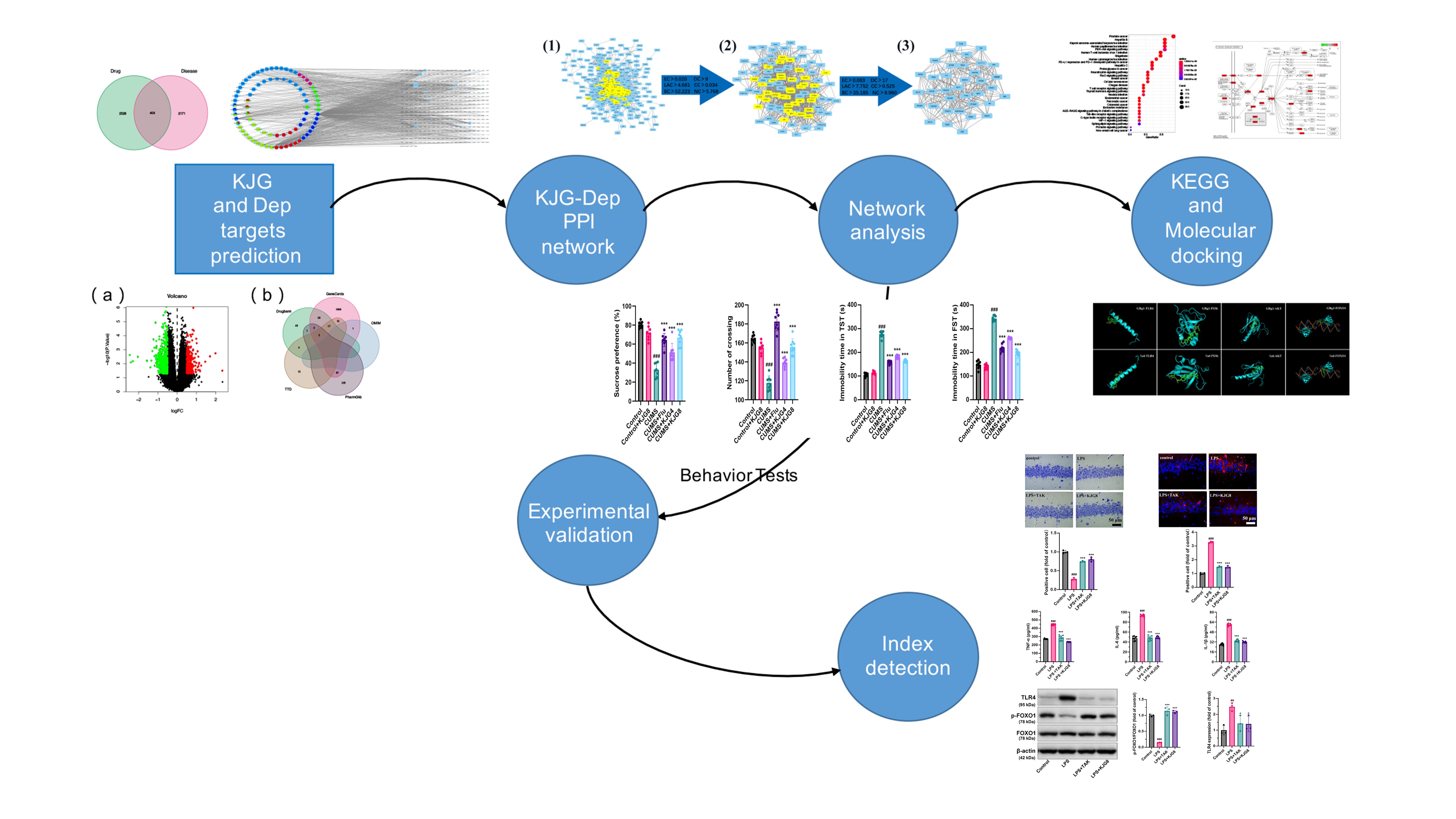


**Fig. S1** Flow chart of the present study.


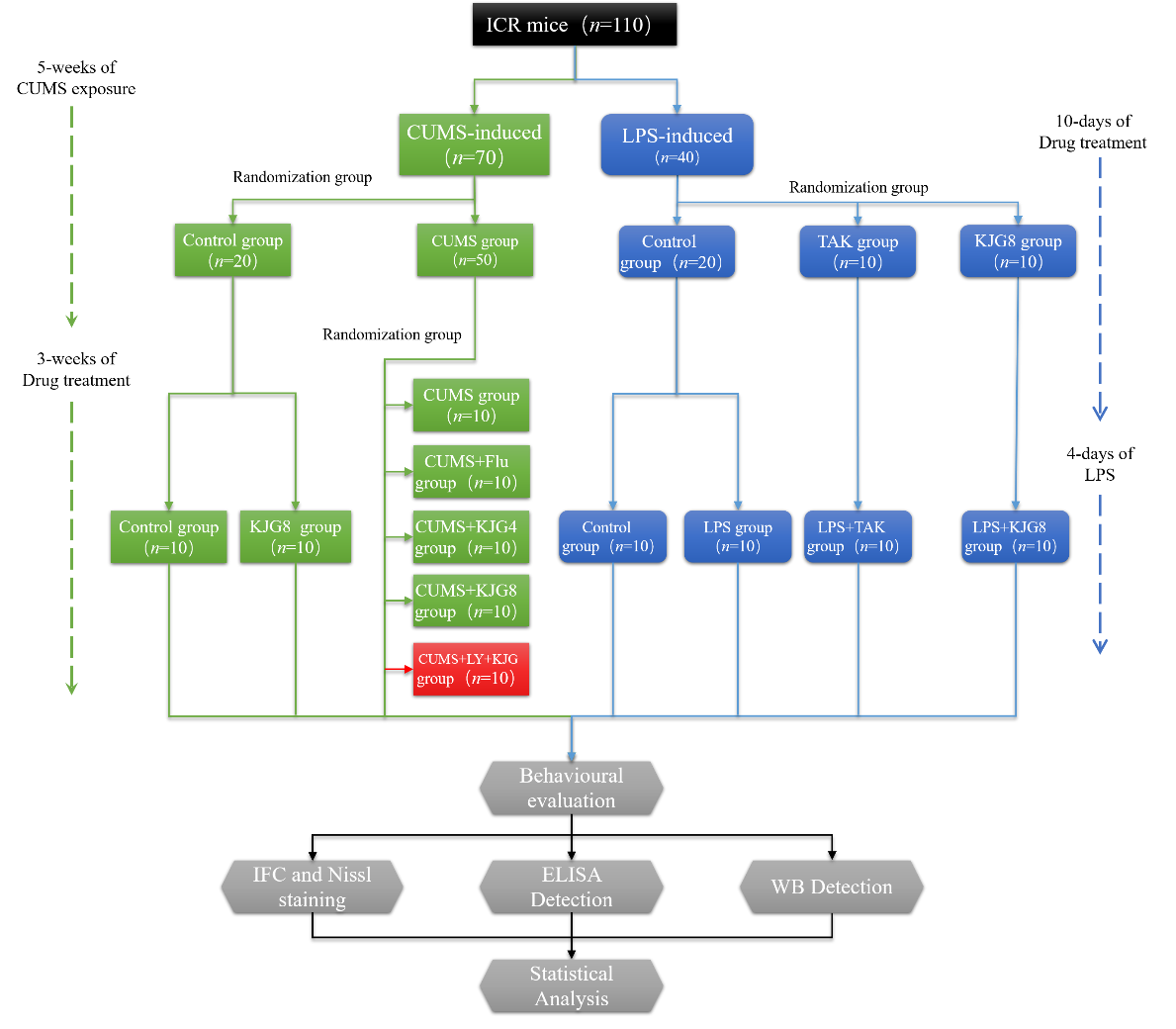


**Fig. S2** Flow chart of the experiments *in vivo.*


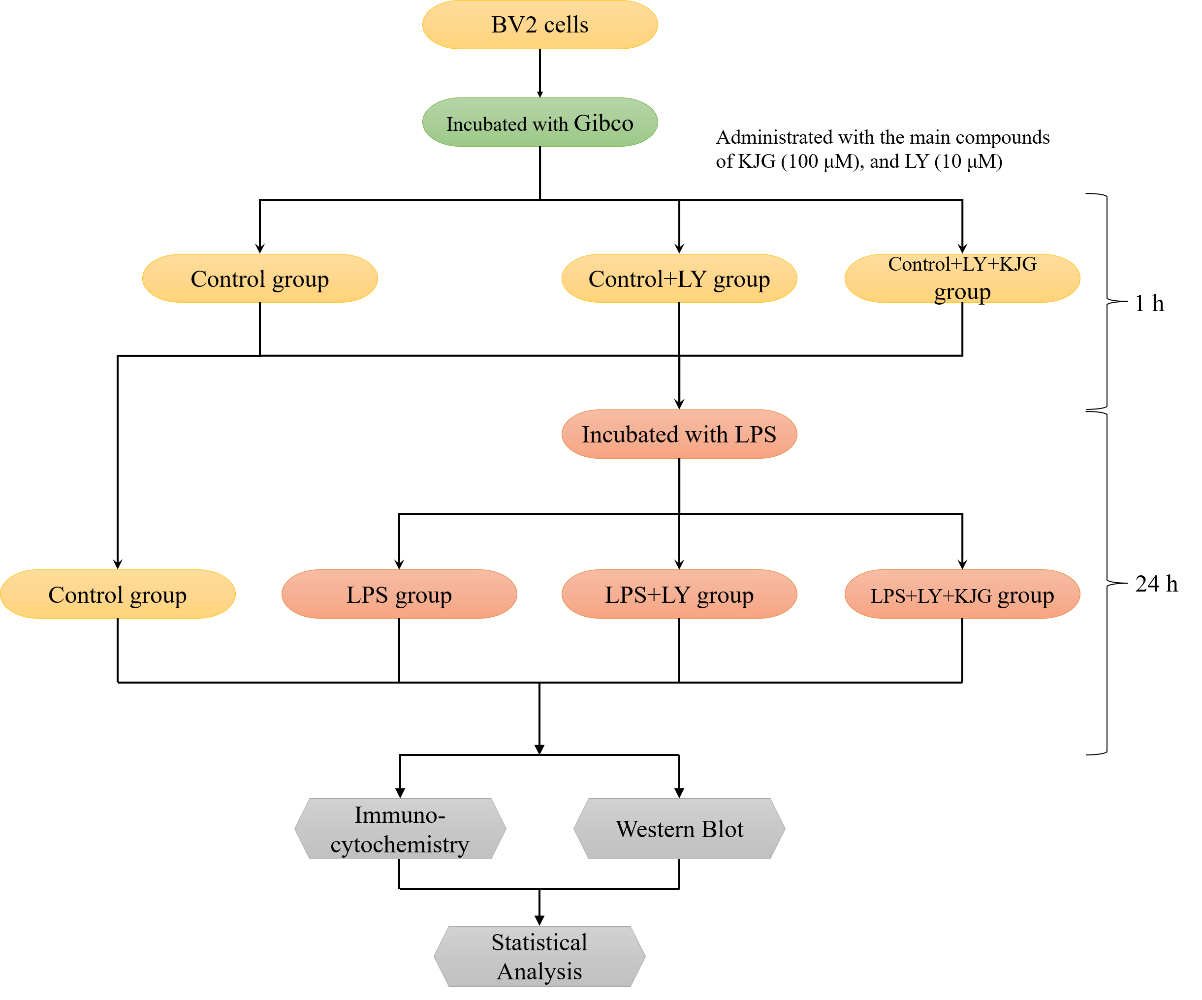


**Fig. S3** Flow chart of the experiments *in vitro*.

**Table S1.** The CCK8 results of Ginsenoside Rg1

| Group (μmol/L) | OD (450 nm) | F value | P value | IC50  (μmol/L) |
| --- | --- | --- | --- | --- |
| Blank | 0.18±0.003 | F (5, 12) = 1.301 | ＜0.001 | 225.618 |
| Control (0) | 0.99±0.001 |  |  |  |
| 50 | 0.84±0.006^***^ |  |  |  |
| 100 | 0.69±0.005^***^ |  |  |  |
| 200 | 0.58±0.015^***^ |  |  |  |
| 500 | 0.48±0.014^***^ |  |  |  |

Note: Values are expressed as mean ± SD (n = 10), **P*<0.05, ***P*<0.01, ****P*<0.001 vs Control group.

**Table S2.** The CCK8 results of Saikosaponin D

| Group (μmol/L) | OD (450 nm) | F value | P value | IC50  (μmol/L) |
| --- | --- | --- | --- | --- |
| Blank | 0.20±0.002 | F (5, 12) = 1.070 | ＜0.01 | 34.364 |
| Control (0) | 0.97±0.013 |  |  |  |
| 10 | 0.84±0.007^***^ |  |  |  |
| 20 | 0.75±0.035^***^ |  |  |  |
| 40 | 0.53±0.004^***^ |  |  |  |
| 80 | 0.39±0.010^***^ |  |  |  |

Note: Values are expressed as mean ± SD (n = 10), **P*<0.05, ***P*<0.01, ****P*<0.001 vs Control group.

**Table S3.** The screened targets of KJG

| ID | Source | Name | ID | Source | Name | ID | Source | Name |
| --- | --- | --- | --- | --- | --- | --- | --- | --- |
| MOL000009 | Bajitian | Vitamin C | MOL000106 | Fuling | Pachypodol | MOL000333 | Gancao | Narwedine |
| MOL000005 | Bajitian | Ohioensin A | MOL000110 | Fuling | Poricoic Acid B | MOL000394 | Gancao | Liquoric Acid |
| MOL009537 | Bajitian | americanin A | MOL000111 | Fuling | Ergotamine | MOL000351 | Gancao | 3'-Methoxyglabridin |
| MOL100009 | Bajitian | Nistose | MOL000098 | Fuling | Pachymic Acid | MOL000375 | Gancao | Gancaonin B |
| MOL004359 | Bajitian, Chishao,Gancao | sitosterol | MOL900279 | Fuling | Cerevisterol | MOL000350 | Gancao | 2,4,4'-Trihydroxychalcone |
| MOL000022 | Chaihu | N-Nonanol | MOL000095 | Fuling | Ergosterol | MOL000338 | Gancao | Glycyrin |
| MOL000070 | Chaihu | Nootkatone | MOL000100 | Fuling | Beta-Amyrin Acetate | MOL000326 | Gancao | 3,3'-Dimethylquercetin |
| MOL000036 | Chaihu | Stearic Acid | MOL000096 | Fuling | Dehydroeburicoicacid | MOL900417 | Gancao | Calycosin |
| MOL000028 | Chaihu | Pentanol | MOL000094 | Fuling, Renshen | Choline | MOL000347 | Gancao | Glyzaglabrin |
| MOL004653 | Chaihu | (+)-Anomalin | MOL000105 | Fuling, Renshen | Adenine | MOL000356 | Gancao | Licochalcone A |
| MOL004609 | Chaihu | Areapillin | MOL000327 | Gancao | Dimethyl Sebacate | MOL000335 | Gancao | Glycyrrhiza-Flavonol A |
| MOL000031 | Chaihu | Encecalin | MOL000398 | Gancao | Isotrifoliol | MOL005012 | Gancao | Licoagroisoflavone |
| MOL000076 | Chaihu | Myrtanol | MOL000385 | Gancao | Ganoderic Acid A | MOL004828 | Gancao | Glepidotin A |
| MOL000030 | Chaihu | Sainfuran | MOL000313 | Gancao | 18alpha-Glycyrrhetinic Acid | MOL100003 | Gancao | Glycyrrhizic acid Ammonium salt |
| MOL000075 | Chaihu | Guaiacol | MOL000334 | Gancao | Tetrahydroharmine | MOL100004 | Gancao | Liquiritin |
| MOL000050 | Chaihu | Vanillin Acetate | MOL000341 | Gancao | Gancaonin I | MOL005348 | Renshen | Ginsenoside-Rh4_qt |
| MOL000051 | Chaihu | Pulegone | MOL004811 | Gancao | Glyasperin C | MOL000232 | Renshen | Malonylginsenoside Rc |
| MOL000084 | Chaihu | Saikosaponin T | MOL004912 | Gancao | Glabrone | MOL000275 | Renshen | Riboflavine |
| MOL000035 | Chaihu | Lauric Acid | MOL000315 | Gancao | Methyl Linoleate | MOL000184 | Renshen | Eicosane |
| MOL000020 | Chaihu | Linolenic Acid | MOL004915 | Gancao | Eurycarpin A | MOL000284 | Renshen | Deoxyharringtonine |
| MOL000047 | Chaihu | Saikosaponin D | MOL000401 | Gancao | 3-Methyl-6,7,8-Trihydropyrrolo[1,2-A]Pyrimidin-2-One | MOL000272 | Renshen | Palmitoleic Acid |
| MOL000078 | Chaihu | Carvone | MOL000308 | Gancao | Glycyrrhetinic Acid | MOL000249 | Renshen | Dianthoside |
| MOL000068 | Chaihu | Thymonin | MOL000382 | Gancao | Isoliquiritigenin | MOL000235 | Renshen | Dianthramine |
| MOL000012 | Chaihu | Alpha-Linolenic Acid | MOL004824 | Gancao | (2S)-6-(2,4-dihydroxyphenyl)-2-(2-hydroxypropan-2-yl)-4-methoxy-2,3-dihydrofuro[3,2-g]chromen-7-one | MOL000303 | Renshen | Protopanaxatriol |
| MOL000041 | Chaihu | Saikosaponin C | MOL000346 | Gancao | Lupiwighteone | MOL000256 | Renshen | Beta-Bisabolene |
| MOL000018 | Chaihu | 8-Nonenoic Acid | MOL004866 | Gancao | 2-(3,4-dihydroxyphenyl)-5,7-dihydroxy-6-(3-methylbut-2-enyl)chromone | MOL000278 | Renshen | 1-Tetradecanol |
| MOL000062 | Chaihu | Adonitol | MOL000357 | Gancao | Hispaglabridin B | MOL000189 | Renshen | Calarene |
| MOL004718 | Chaihu | α-spinasterol | MOL000500 | Gancao | Vestitol | MOL000255 | Renshen | Dibutyl Phthalate |
| MOL000029 | Chaihu | Pulsatillic Acid | MOL004957 | Gancao | HMO | MOL000301 | Renshen | Adenosine Triphosphate |
| MOL000085 | Chaihu | 2-Methylcyclopentanone | MOL004808 | Gancao | glyasperin B | MOL000219 | Renshen | Vitamin B12 |
| MOL000088 | Chaihu | Linalool | MOL000396 | Gancao | Methyl-24-Hydroxy-11-Deoxoglycyrrhetate | MOL000271 | Renshen | Widdrol |
| MOL000046 | Chaihu | Octalupine | MOL004980 | Gancao | Inflacoumarin A | MOL000171 | Renshen | Ginsenoside F1 |
| MOL000083 | Chaihu | Nonanoic Acid | MOL000345 | Gancao | Isoglycyrol | MOL000207 | Renshen | 1-Heptadecanol |
| MOL000081 | Chaihu | Saikosaponin A | MOL004883 | Gancao | Licoisoflavone | MOL000167 | Renshen | D-Mannuronic Acid |
| MOL000016 | Chaihu | Tetradecane | MOL000228 | Gancao | Gamma-Sitosterol | MOL000287 | Renshen | 3,5-Dimethyl-4-Methoxybenzoic Acid |
| MOL000045 | Chaihu | Linoleyl Acetate | MOL000403 | Gancao | Tetrahydropalmatine | MOL000258 | Renshen | Protopanaxadiol |
| MOL000039 | Chaihu | Caprylic Acid | MOL003896 | Gancao | 7-Methoxy-2-methyl isoflavone | MOL000160 | Renshen | Alpha-Cadinol |
| MOL000048 | Chaihu | 7-Octen-4-Ol | MOL000366 | Gancao | Licobenzofuran | MOL000191 | Renshen | Dauricine |
| MOL000043 | Chaihu | Phenylacetic Acid | MOL000381 | Gancao | Licocoumarone | MOL005321 | Renshen | Frutinone A |
| MOL000060 | Chaihu | Myrtenal | MOL000316 | Gancao | 4'-O-Methylglabridin | MOL000190 | Renshen | Hexadecanoic Acid |
| MOL100001 | Chaihu | Saikosaponin a | MOL000354 | Gancao | Licoricidin | MOL000186 | Renshen | Malvic Acid |
| MOL100002 | Chaihu | Saikosaponin d | MOL000343 | Gancao | Methylglyoxal | MOL000239 | Renshen | Ginsenoside La |
| MOL000064 | Chaihu, Gancao | Quercetin | MOL004991 | Gancao | 7-Acetoxy-2-methylisoflavone | MOL000261 | Renshen | Tauremisin |
| MOL050354 | Chaihu, Gancao | isorhamnetin | MOL004990 | Gancao | 7,2',4'-trihydroxy－5-methoxy-3－arylcoumarin | MOL000180 | Renshen | Citronellal |
| MOL000017 | Chaihu, Renshen | Methyl Palmitate | MOL000377 | Gancao | Glyuranolide | MOL000279 | Renshen | Trifolirhizin |
| MOL000059 | Chaihu, Renshen | Tridecanoic Acid | MOL000348 | Gancao | Glycyrrhetol | MOL000166 | Renshen | Aposiopolamine |
| MOL000052 | Chaihu, Renshen, Chishao | Stigmasterol | MOL000390 | Gancao | Umbelliferone | MOL000254 | Renshen | Vitamin B5 |
| MOL000061 | Chaihu, Renshen, Gancao | Kaempferol | MOL000311 | Gancao | Corylifolinin | MOL000174 | Renshen | 20(S)-Protopanaxadiol |
| MOL000151 | Chishao | Paeoniflorigenone | MOL004961 | Gancao | Quercetin der. | MOL000165 | Renshen | Biotin |
| MOL000152 | Chishao | Catechin | MOL000379 | Gancao | 5,6,7,8-Tetrahydro-4-Methylquinoline | MOL000241 | Renshen | Guanosine |
| MOL000153 | Chishao | Epigallocatechin | MOL004849 | Gancao | 3-(2,4-dihydroxyphenyl)-8-(1,1-dimethylprop-2-enyl)-7-hydroxy-5-methoxy-coumarin | MOL000066 | Renshen | Beta-Humulene |
| MOL000144 | Chishao | Gallocatechin | MOL000371 | Gancao | Phaseollinisoflavan | MOL000229 | Renshen | Pentadecanoic Acid |
| MOL000073 | Chishao | Spinasterol | MOL000367 | Gancao | Formononetin | MOL000157 | Renshen | Argininyl-Fructosyl-Glucose |
| MOL000143 | Chishao | (+)-Catechin | MOL000336 | Gancao | Gancaonin A | MOL000188 | Renshen | Adenosine |
| MOL000137 | Chishao | Lactiflorin | MOL000399 | Gancao | Liquiritigenin | MOL000304 | Renshen | Ginsenoside-La |
| MOL001002 | Chishao | ellagic acid | MOL002565 | Gancao | Medicarpin | MOL000246 | Renshen | P-Glucosyloxymandelonitrile |
| MOL000146 | Chishao | (-)-Catechin | MOL000322 | Gancao | Isoramanone | MOL000257 | Renshen | 16-Oxoseratenediol |
| MOL002714 | Chishao | baicalein | MOL900239 | Gancao | Jaranol | MOL000185 | Renshen | Ginsenol |
| MOL000140 | Chishao | Albiflorin | MOL000397 | Gancao | Hispidulin | MOL000282 | Renshen | Malonylginsenoside Rd |
| MOL100008 | Chishao | Paeoniflorin | MOL000352 | Gancao | Alpha-Trihydroxy Coprostanic Acid | MOL000274 | Renshen | Mannose |
| MOL000154 | Chishao, Renshen | Daucosterol | MOL000376 | Gancao | Methyl-24-Hydroxyglycyrrhetate | MOL000193 | Renshen | Protopine |
| MOL000104 | Fuling | Hydrangeic Acid | MOL005000 | Gancao | Gancaonin G | MOL000202 | Renshen | Epsilon-Cadinene |
| MOL000097 | Fuling | Porphyroxine | MOL000331 | Gancao | 2,5-Dihydroxymethyl-3,4-Dihydroxypyrrolidine | MOL000168 | Renshen | Pandamine |
| MOL900275 | Fuling | trametenolic acid | MOL000307 | Gancao | 18beta-Glycyrrhetinic Acid | MOL100005 | Renshen | Ginsenoside Rg1 |
| MOL900273 | Fuling | (2R)-2-[(3S,5R,10S,13R,14R,16R,17R)-3,16-dihydroxy-4,4,10,13,14-pentamethyl-2,3,5,6,12,15,16,17-octahydro-1H-cyclopenta[a]phenanthren-17-yl]-6-methylhept-5-enoic acid | MOL005016 | Gancao | Odoratin | MOL100006 | Renshen | Ginsenoside Rb1 |
| MOL000109 | Fuling | O-Acetylpachymic Acid-25-Ol | MOL000344 | Gancao | Hispaglabridin A | MOL100007 | Renshen | Ginsenoside Re |
| MOL000107 | Fuling | Eburicol | MOL004864 | Gancao | 5,7-dihydroxy-3-(4-methoxyphenyl)-8-(3-methylbut-2-enyl)chromone |  |  |  |

**Table S4.** The riched genes in the top 15 KEGG terms via enrichment analysis

| ID | Description | GeneRatio | qvalue | Gene ID |
| --- | --- | --- | --- | --- |
| hsa05215 | Prostate cancer | 25/36 | 1.93E-39 | PIK3CA, PIK3R2, NFKBIA, MTOR, CTNNB1, PDPK1, RELA, NFKB1, HSP90AA1, MAPK1, AKT1, CREBBP, NRAS, TP53, EP300, PIK3CB, PIK3R1, RB1, EGFR, EGF, AKT3, AKT2, FOXO1, AR, PTEN |
| hsa05161 | Hepatitis B | 23/36 | 1.12E-29 | TNF, PIK3CA, PIK3R2, NFKBIA, MAPK14, JUN, RELA, TLR4, NFKB1, MAPK1, MYC, AKT1, CREBBP, MAPK8, NRAS, TP53, EP300, PIK3CB, TRAF6, PIK3R1, RB1, AKT3, AKT2 |
| hsa05167 | Kaposi sarcoma-associated herpesvirus infection | 23/36 | 5.18E-28 | PIK3CA, PIK3R2, NFKBIA, MTOR, MAPK14, CTNNB1, JUN, RELA, NFKB1, MAPK1, MYC, AKT1, CREBBP, VEGFA, MAPK8, NRAS, TP53, EP300, PIK3CB, PIK3R1, RB1, AKT3, AKT2 |
| hsa05165 | Human papillomavirus infection | 23/36 | 3.41E-23 | TNF, PIK3CA, PIK3R2, MTOR, CTNNB1, RELA, NFKB1, MAPK1, AKT1, CREBBP, VEGFA, NRAS, TP53, EP300, PIK3CB, PIK3R1, RB1, EGFR, EGF, AKT3, AKT2, FOXO1, PTEN |
| hsa04151 | PI3K-AKT signaling pathway | 23/36 | 1.26E-22 | PIK3CA, PIK3R2, MTOR, PDPK1, IL2, RELA, TLR4, NFKB1, MAPK1, MYC, AKT1, VEGFA, NRAS, TP53, PIK3CB, PIK3R1, EGFR, EGF, AKT3, AKT2, PTEN, FOXO1 |
| hsa05166 | Human T-cell leukemia virus 1 infection | 22/36 | 3.29E-25 | TNF, PIK3CA, PIK3R2, NFKBIA, JUN, IL2, RELA, NFKB1, MAPK1, MYC, AKT1, CREBBP, MAPK8, NRAS, TP53, EP300, PIK3CB, PIK3R1, RB1, AKT3, AKT2, PTEN |
| hsa05131 | Shigellosis | 22/36 | 2.61E-24 | TNF, PIK3CA, PIK3R2, NFKBIA, MTOR, MAPK14, JUN, RELA, TLR4, NFKB1, MAPK1, AKT1, MAPK8, TP53, PIK3CB, TRAF6, PIK3R1, EGFR, AKT3, AKT2, FOXO1 |
| hsa05163 | Human cytomegalovirus infection | 21/36 | 1.52E-23 | TNF, PIK3CA, PIK3R2, NFKBIA, MTOR, MAPK14, CTNNB1, RELA, NFKB1, MAPK1, MYC, AKT1, VEGFA, NRAS, TP53, PIK3CB, PIK3R1, RB1, EGFR, AKT3, AKT2 |
| hsa05235 | PD-L1 expression and PD-1 checkpoint pathway in cancer | 20/36 | 1.12E-29 | PIK3CA, PIK3R2, NFKBIA, MTOR, MAPK14, JUN, RELA, TLR4, NFKB1, MAPK1, AKT1, NRAS, PIK3CB, TRAF6, PIK3R1, EGFR, EGF, AKT3, AKT2, PTEN |
| hsa05160 | Hepatitis C | 20/36 | 7.10E-25 | TNF, PIK3CA, PIK3R2, NFKBIA, CTNNB1, RELA, NFKB1, MAPK1, MYC, AKT1, NRAS, TP53, PIK3CB, TRAF6, PIK3R1, RB1, EGFR, EGF, AKT3, AKT2 |
| hsa05205 | Proteoglycans in cancer | 20/36 | 7.55E-23 | TNF, PIK3CA, PIK3R2, MTOR, MAPK14, CTNNB1, PDPK1, TLR4, MAPK1, MYC, AKT1, VEGFA, NRAS, TP53, PIK3CB, PIK3R1, ESR1, EGFR, AKT3, AKT2 |
| hsa04722 | Neurotrophin signaling pathway | 19/36 | 2.89E-25 | PIK3CA, PIK3R2, NFKBIA, MAPK14, PDPK1, JUN, RELA, NFKB1, MAPK1, AKT1, MAPK8, NRAS, TP53, PIK3CB, TRAF6, PIK3R1, AKT3, AKT2 |
| hsa04068 | FoxO signaling pathway | 19/36 | 1.01E-24 | PIK3CA, PIK3R2, MAPK14, TLR4, MAPK1, AKT1, CREBBP, MAPK8, NRAS, EP300, PIK3CB, PIK3R1, EGFR, EGF, AKT3, AKT2, FOXO1, PTEN |
| hsa05224 | Breast cancer | 19/36 | 8.16E-24 | PIK3CA, PIK3R2, MTOR, CTNNB1, JUN, MAPK1, MYC, AKT1, NRAS, TP53, PIK3CB, PIK3R1, RB1, ESR1, EGFR, EGF, AKT3, AKT2, PTEN |
| hsa04218 | Cellular senescence | 19/36 | 2.05E-23 | PIK3CA, PIK3R2, MTOR, MAPK14, RELA, NFKB1, MAPK1, MYC, AKT1, NRAS, TP53, PIK3CB, PIK3R1, RB1, AKT3, AKT2, FOXO1, PTEN |
